# Early Neuroprotective Effects of Erucic Acid and Gentisic Acid on Hippocampal Glutamate Concentrations and Alzheimer-like Molecular Alterations in an Intracerebroventricular Streptozotocin Rat Model

**DOI:** 10.3390/ph19091392

**Published:** 2026-09-02

**Authors:** İbrahim Gecili, Muhammed Sait Ertuğrul, Irmak Ferah Okkay, Onur Şenol, Ufuk Okkay, Mustafa Özkaraca, Ziadoon Al-Yaqoobi, Cemil Bayram, A. M. Abd El-Aty, Ali Taghizadehghalehjoughi, Ahmet Hacımüftüoğlu

**Affiliations:** 1 Department of Medical Pharmacology, Faculty of Medicine, Beykent University, Istanbul 34398, Türkiye; 2 Department of Food, Feed and Medicine, Hemp Research Institute, Ondokuz Mayis University, Samsun 55270, Türkiye; sait.ertugrul@omu.edu.tr; 3 Department of Medical Pharmacology, Faculty of Medicine, Ataturk University, Erzurum 25240, Türkiye; irmakferah@atauni.edu.tr (I.F.O.); ufukokkay@gmail.com (U.O.); ziadoon.azhar24@gmail.com (Z.A.-Y.); abdelaty44@hotmail.com (A.M.A.E.-A.); hacimuftuoglu@gmail.com (A.H.); 4 Department of Analytical Chemistry, Faculty of Pharmacy, Ataturk University, Erzurum 25240, Türkiye; onursenol@atauni.edu.tr; 5 Department of Pathology, Faculty of Veterinary Medicine, Sivas Cumhuriyet University, Sivas 58140, Türkiye; mustafaozkaraca@cumhuriyet.edu.tr; 6 Department of Veterinary Pharmacology and Toxicology, Faculty of Veterinary Medicine, Ataturk University, Erzurum 25240, Türkiye; cemil489@gmail.com; 7 Department of Pharmacology, Faculty of Veterinary Medicine, Cairo University, Giza 12211, Egypt; 8 Department of Medical Pharmacology, Faculty of Medicine, Bilecik Şeyh Edebali University, Bilecik 11230, Türkiye; ali.tgzd@bilecik.edu.tr

**Keywords:** Alzheimer’s disease model, erucic acid, gentisic acid, hippocampal tissue glutamate, intracerebroventricular streptozotocin, early neuroprotection

## Abstract

**Background:** Alterations in glutamatergic homeostasis have been implicated in Alzheimer’s disease, but the relationship between total hippocampal tissue glutamate and Alzheimer-like molecular alterations remains unclear. This study evaluated the early neuroprotective potential of gentisic acid (GA) and erucic acid (EA) in an intracerebroventricular streptozotocin (icv-STZ)-induced rat model of sporadic Alzheimer-like pathology. **Methods:** Sixty-four female Sprague–Dawley rats were randomly assigned to eight groups: control, sham, STZ, STZ + GA (100 or 200 mg/kg/day), STZ + EA (25 or 50 mg/kg/day), and STZ + memantine (10 mg/kg/day). Treatments began immediately after STZ and continued orally for 21 days. Behavioral performance was assessed by Morris water maze and passive avoidance tests. Total hippocampal tissue glutamate was quantified by LC–MS/MS. Neuronal degeneration was assessed histopathologically, whereas tau, amyloid-β, and AChE immunoreactivity and tau-, APP-, and AChE-related in situ hybridization signals were evaluated. **Results:** Compared with controls, STZ-treated rats exhibited poorer behavioral performance, higher total hippocampal tissue glutamate, greater neuronal degeneration, and increased molecular signals. GA and EA administration was associated with attenuation of these alterations, with generally stronger effects at higher doses. EA at 50 mg/kg/day showed the lowest mean total hippocampal tissue glutamate among treatment groups. GA at 200 mg/kg/day and EA at 50 mg/kg/day showed the most consistent preservation across behavioral, histopathological, and molecular endpoints. **Conclusions:** Early GA and EA administration was associated with neuroprotective effects in the icv-STZ model. Because treatment began immediately after STZ administration, the findings support early or preventive neuroprotection rather than reversal of established Alzheimer-like pathology. Total tissue glutamate measurements neither distinguish extracellular glutamate nor directly demonstrate glutamate-mediated excitotoxicity.

## 1. Introduction

Alzheimer’s disease (AD) is a progressive neurodegenerative disorder responsible for 60–70% of all dementia cases worldwide, with the global prevalence projected to triple by 2050 [1,2]. Despite decades of intensive research, AD is characterized by insidious cognitive decline and memory impairment and lacks a disease-modifying therapy [3].

The neuropathological hallmarks of AD include extracellular deposition of amyloid-β (Aβ) plaques, intraneuronal accumulation of hyperphosphorylated tau neurofibrillary tangles, synaptic loss, and progressive neuronal degeneration predominantly affecting the hippocampus and cerebral cortex [4,5]. These changes are accompanied by sustained neuroinflammation and mitochondrial dysfunction, with the cholinergic and glutamatergic neurotransmitter systems playing central roles in both the pathophysiology and treatment of the disease [6,7].

According to the cholinergic hypothesis, loss of cholinergic neurons in the basal forebrain and reduced acetylcholine synthesis directly contribute to the cognitive deficits observed in AD [8,9]. Acetylcholinesterase (AChE), the enzyme responsible for acetylcholine hydrolysis at the synaptic cleft, is overexpressed in AD and colocalizes with Aβ plaques, thereby amplifying neurotoxic cascades [10,11]. Glutamatergic excitotoxicity, in turn, results from the pathological accumulation of extracellular glutamate, leading to the sustained overstimulation of N-methyl-D-aspartate (NMDA) receptors, excessive calcium influx, and neuronal death [12,13]. Hippocampal CA1 pyramidal neurons are particularly vulnerable to this insult [14]. Impaired astrocytic glutamate reuptake further exacerbates synaptic dysfunction by promoting extrasynaptic glutamate accumulation [15,16,17]. The therapeutic relevance of the glutamatergic pathway is clinically supported by memantine, an approved NMDA receptor antagonist used for the symptomatic treatment of moderate-to-severe AD, which was included as a positive control in the present study [18,19,20].

The intracerebroventricular streptozotocin (icv-STZ) rat model is a widely used nontransgenic model that reproduces selected metabolic, cognitive, and neuropathological alterations relevant to sporadic Alzheimer-like pathology. Although dementia is a broader clinical syndrome, the icv-STZ model is used specifically to investigate metabolic, cognitive, and neurodegenerative alterations relevant to sporadic Alzheimer-like pathology. This model reproduces cognitive impairment, Aβ deposition, tau hyperphosphorylation, cholinergic deficits, and oxidative stress within a tractable experimental timeline and has been widely used for the evaluation of candidate neuroprotective agents [21,22,23]. Although the icv-STZ model reproduces selected metabolic, cognitive, and neurodegenerative alterations relevant to sporadic Alzheimer-like pathology, it does not capture the full complexity, progressive course, or clinical presentation of human AD.

In this context, naturally occurring compounds with multitarget neuroprotective effects have attracted increasing research interest. Gentisic acid (GA; 2,5-dihydroxybenzoic acid) is a phenolic metabolite of aspirin with well-documented antioxidant, anti-inflammatory, and antiapoptotic properties [24]. GA has been reported to suppress oxidative stress and neuroinflammation and to exert neuroprotective effects in experimental neurodegeneration models [25]. Recent evidence has shown that GA improves cognitive performance and attenuates acetylcholinesterase activity, amyloid-β accumulation, hyperphosphorylated tau, oxidative stress, and neuroinflammatory markers in an experimental Alzheimer-like model [26]. Thus, the general neuroprotective effects of GA in experimental Alzheimer-like pathology have prior support. However, GA has not been adequately characterized in the icv-STZ model using LC–MS/MS-based quantification of total hippocampal tissue glutamate integrated with behavioral, histopathological, and molecular assessments. Erucic acid (EA; cis-13-docosenoic acid) is a long-chain monounsaturated fatty acid and a recognized ligand of peroxisome proliferator-activated receptor delta (PPAR-δ). PPAR-δ activation has been reported to suppress neuroinflammation and confer neuroprotection in transgenic AD models [27,28], while the memory-enhancing effects of EA have been documented in a cholinergic deficit model [29]. Previous studies provide support for the memory-enhancing and neuroprotective potential of EA [29,30,31]. Pharmacological modulation of glutamate transporter activity has also been demonstrated in other experimental neurobehavioral models [32]. However, the effects of EA in the icv-STZ model, particularly in relation to total hippocampal tissue glutamate concentrations, remain insufficiently characterized.

The present study was designed to evaluate the early neuroprotective potential of gentisic acid and erucic acid in an icv-STZ-induced rat model of sporadic Alzheimer-like pathology, with memantine included as a positive control. The exclusive use of female Sprague–Dawley rats was based on epidemiological evidence indicating a higher AD burden in women and experimental findings suggesting sex-related interactions in neuroprotective responses [2]. Neuroprotection was assessed through a multiendpoint approach encompassing spatial and associative learning and memory (Morris water maze and passive avoidance tests), LC–MS/MS-based quantification of total hippocampal tissue glutamate, histopathological analysis, and immunohistochemical and in situ hybridization analyses of tau, Aβ, AChE, and APP-related expression patterns. Accordingly, the principal contribution of the present study lies in the comparative evaluation of GA and EA in the icv-STZ model by integrating LC–MS/MS-based quantification of total hippocampal tissue glutamate with behavioral, histopathological, immunohistochemical, and in situ hybridization assessments, rather than in the first demonstration of their general neuroprotective effects.

## 2. Results

### 2.1. Behavioral Test Results

#### 2.1.1. Passive Avoidance Test

Passive avoidance performance was assessed by measuring step-through latency during two post-treatment sessions conducted on consecutive days. All animals had undergone foot-shock-based passive avoidance conditioning before icv-STZ or vehicle administration. Group-level summary statistics for each assessment session are presented in Table 1. As several groups reached the predefined 300-s cut-off, the results were interpreted in consideration of the associated ceiling effect. Differences between the group means across the two sessions are reported for descriptive purposes only and were not subjected to inferential statistical analysis.

On post-treatment Day 1, the STZ group exhibited a significantly shorter step-through latency than the control group (*p* < 0.01). Compared with the STZ group, significantly longer latencies were observed in the sham, GA200, EA25, EA50, and memantine groups, whereas no significant difference was detected for the GA100 group.

On post-treatment Day 2, the STZ group continued to exhibit a significantly shorter step-through latency than the control group (*p* < 0.05). The sham, GA200, and EA50 groups reached the 300-s cut-off and exhibited significantly longer latencies than the STZ group (*p* < 0.01). Significantly longer latencies were also observed in the EA25 and memantine groups (*p* < 0.05), whereas the GA100 group did not differ significantly from the STZ group.

Mean changes were calculated from the group means as Day 2 minus Day 1 and are presented for descriptive purposes only. The control group reached the 300-s cut-off on both post-treatment assessment days, producing a ceiling effect that precluded the detection of any further increase. In contrast, the sham group increased from 272.80 ± 33.00 s on Day 1 to 300.00 ± 0.00 s on Day 2. Collectively, these findings indicate reduced passive avoidance performance following icv-STZ administration and relative preservation of conditioned avoidance behavior, particularly in the GA200 and EA50 groups.

#### 2.1.2. Morris Water Maze Results

**Figure 1:**
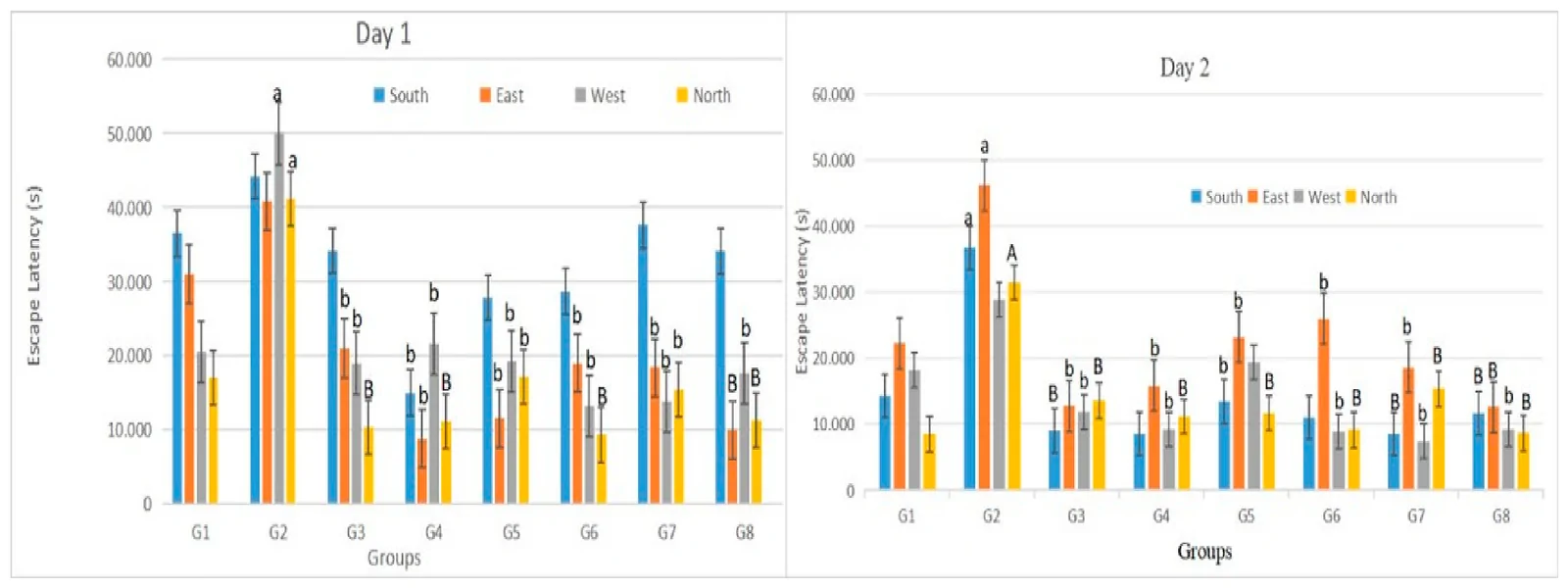
Morris water maze escape latency results on Days 1 and 2 of the acquisition phase. The experimental groups were as follows: G1, control; G2, STZ (3 mg/kg, i.c.v.); G3, sham (i.c.v. citrate buffer); G4, STZ + GA (100 mg/kg/day); G5, STZ + GA (200 mg/kg/day); G6, STZ + EA (25 mg/kg/day); G7, STZ + EA (50 mg/kg/day); and G8, STZ + MEM (10 mg/kg/day). GA, EA, and MEM were administered orally for 21 consecutive days following STZ injection. Statistically significant differences are indicated as follows: ^a^ *p* < 0.05 and ^A^ *p* < 0.01 versus the control group; ^b^ *p* < 0.05 and ^B^ *p* < 0.01 versus the STZ group. GA, gentisic acid; EA, erucic acid; MEM, memantine hydrochloride; STZ, streptozotocin; i.c.v., intracerebroventricular.

In the Morris water maze test, escape latency, defined as the time required to locate the hidden platform, was recorded during the four-day acquisition phase. Escape latency results for Days 1 and 2 are presented in Figure 1.

**Figure 2:**
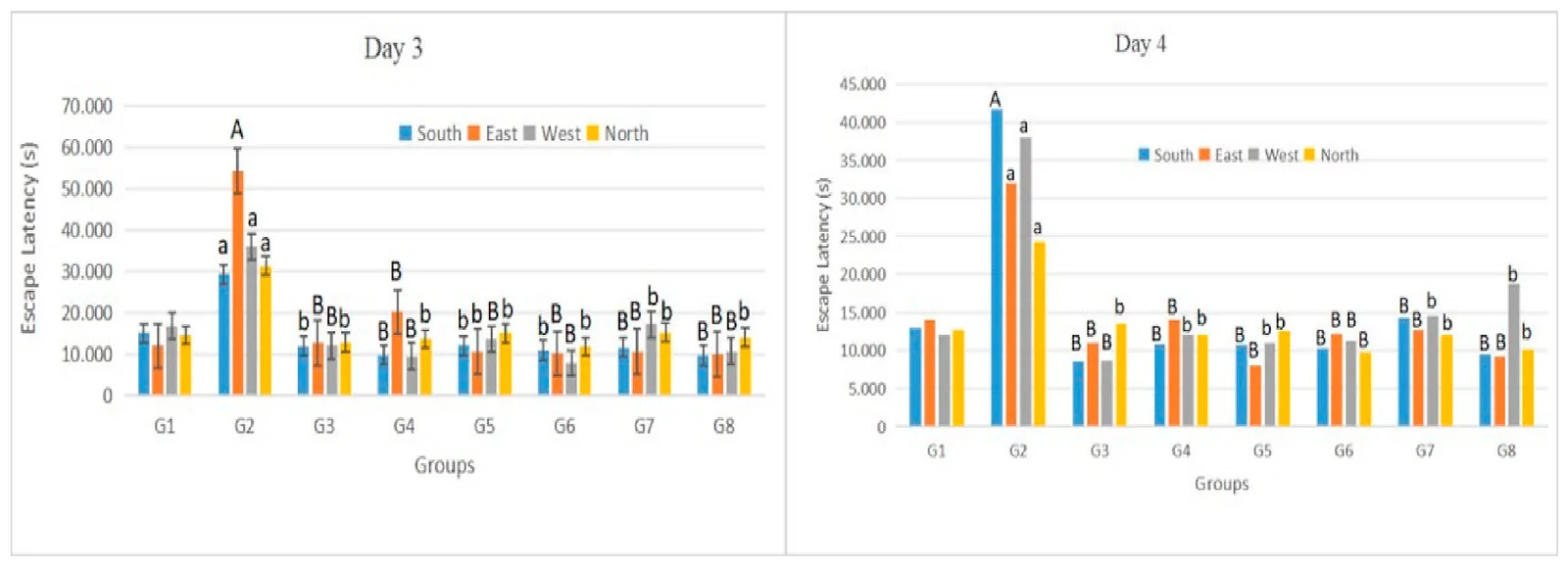
Morris water maze escape latency results on Days 3 and 4 of the acquisition phase. The experimental groups were as follows: G1, control; G2, STZ (3 mg/kg, i.c.v.); G3, sham (i.c.v. citrate buffer); G4, STZ + GA (100 mg/kg/day); G5, STZ + GA (200 mg/kg/day); G6, STZ + EA (25 mg/kg/day); G7, STZ + EA (50 mg/kg/day); and G8, STZ + MEM (10 mg/kg/day). GA, EA, and MEM were administered orally for 21 consecutive days following STZ injection. Statistically significant differences are indicated as follows: ^a^ *p* < 0.05 and ^A^ *p* < 0.01 versus the control group; ^b^ *p* < 0.05 and ^B^ *p* < 0.01 versus the STZ group. GA, gentisic acid; EA, erucic acid; MEM, memantine hydrochloride; STZ, streptozotocin; i.c.v., intracerebroventricular.

Escape latency results for Days 3 and 4 of the acquisition phase are presented in Figure 2.

Representative heatmaps from one animal per group are provided as qualitative visualizations of swimming patterns during the Day 5 probe trial (Supplementary Figure S1).

### 2.2. LC–MS/MS Analysis of Hippocampal Glutamate

Hippocampal glutamate concentrations were determined using LC–MS/MS. Approximately 200 mg of hippocampal tissue was collected from each animal. To minimize variability associated with tissue quantity and extraction conditions, identical tissue weights and extraction volumes were used for all samples. Hippocampal tissue samples were homogenized in 1 mL of acetonitrile for 2 min using stainless-steel beads in a TissueLyser II system. The homogenates were incubated at −20 °C for 1 h and subsequently centrifuged at 12,000 rpm. Following centrifugation, the supernatants were passed through 0.45 µm membrane filters to remove particulate matter and then subjected to quantitative LC–MS/MS analysis.

Analyses were performed using an Agilent 6460 LC–MS/MS system equipped with an Agilent Jet Stream electrospray ionization source operating in positive-ion mode. Glutamic acid was monitored in multiple reaction monitoring (MRM) mode using two precursor-to-product ion transitions: *m*/*z* 147.8 → 129.9 and *m*/*z* 147.8 → 84.0. The transition *m*/*z* 147.8 → 129.9 was used for quantification, whereas *m*/*z* 147.8 → 84.0 served as the qualifier transition. Chromatographic signals attributed to glutamic acid were observed at retention times of approximately 2.0–2.1 min. An ion-ratio tolerance of ±20% relative to the reference standard was used as the predefined identification criterion. The signal observed in the blank did not meet this criterion and was therefore not identified as glutamic acid. Representative MRM chromatograms and product-ion spectra are presented in Supplementary Figure S2.

**Figure 3:**
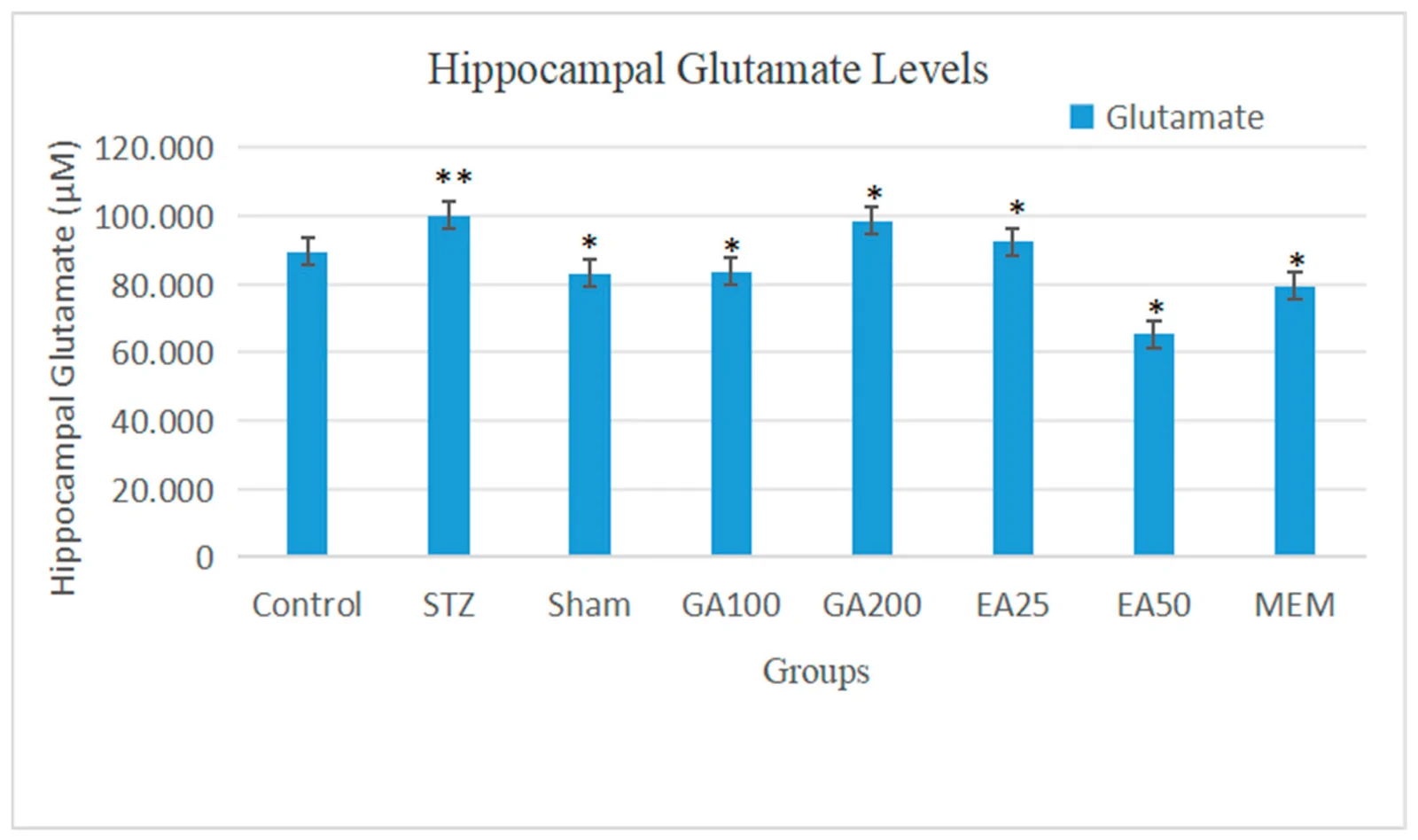
LC–MS/MS analysis of total hippocampal tissue glutamate. Glutamate concentrations in hippocampal tissue homogenates were measured by LC–MS/MS and are presented as mean ± SD. Compared with the control group, the STZ group showed an elevated mean glutamate concentration, whereas EA50 showed the lowest mean concentration among the treatment groups. Statistical significance versus the control group is indicated as follows: * *p* < 0.05 and ** *p* < 0.01.

The nebulizer pressure was set at 35 psi, and the capillary voltage was 4500 V. The injection volume was 5 µL. The mobile phase consisted of deionized water containing 0.1% formic acid and acetonitrile at a ratio of 70:30 (*v*/*v*). Chromatographic separation was performed using an InfinityLab Poroshell 120 SB-AQ column (3.0 × 150 mm, 2.7 µm) maintained at 30 °C. The flow rate was 0.3 mL/min, and the total run time was 4 min. Calibration curves were prepared using reference glutamate standards, and their linearity was confirmed by regression analysis before quantitative analysis of the tissue samples. The LC–MS/MS findings are presented in Figure 3.

### 2.3. Histopathological Evaluation Results

Histopathological evaluation revealed significant differences in neuronal degeneration across the experimental groups in the hippocampal (CA1/CA2, CA3) and cortical regions (*p* < 0.05). The STZ group showed increased degeneration, whereas the treatment groups exhibited reduced degeneration. The results are summarized in Table 2.

The histological architecture in the hippocampal (CA1/CA2, CA3) and cortical regions was normal in the control and sham groups. In contrast, varying degrees of neuronal degeneration were observed in these regions in the STZ and treatment groups.

Degenerative changes were most prominent in the CA3 region, particularly in the STZ group, where severe alterations were detected. In the same group, moderate degeneration was observed in the CA1/CA2 and cortical regions.

In the treatment groups, degenerative changes were generally mild compared with those in the STZ group across all the regions examined.

**Figure 4:**
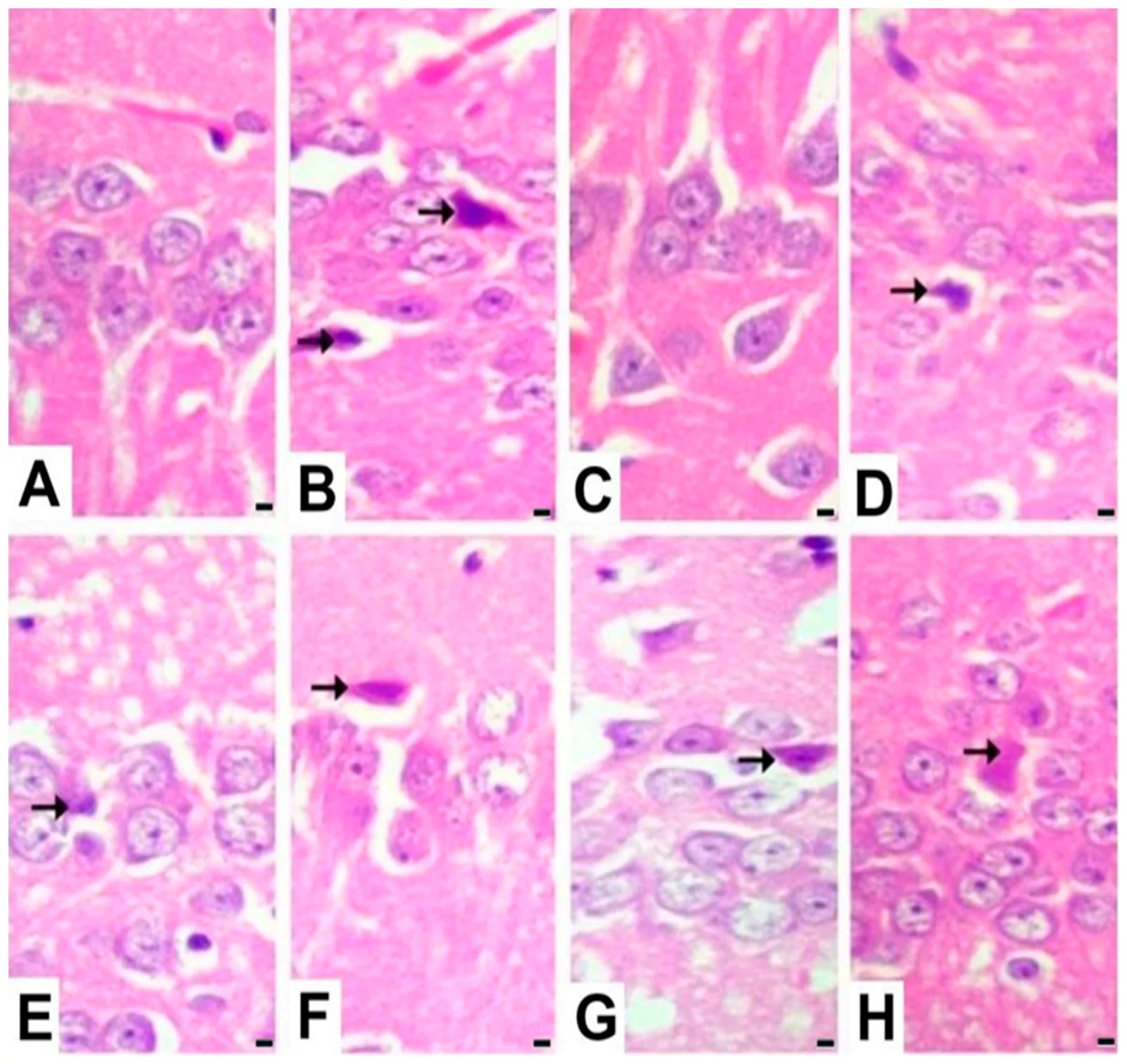
Histopathological changes in the cornu ammonis CA1/CA2 region revealed by hematoxylin and eosin (H&E) staining. (**A**) Control group (G1) showing preserved histological architecture. (**B**) STZ group (G2) showing marked neuronal degeneration. (**C**) Sham group (G3) showing preserved histological architecture without evident neuronal degeneration. (**D**) GA100, (**E**) GA200, (**F**) EA25, (**G**) EA50, and (**H**) memantine groups showing mild neuronal degeneration compared with the STZ group. Degenerative neurons (→) are characterized by cellular shrinkage and hyperchromatic nuclei. Scale bar: 2 µm.

**Figure 5:**
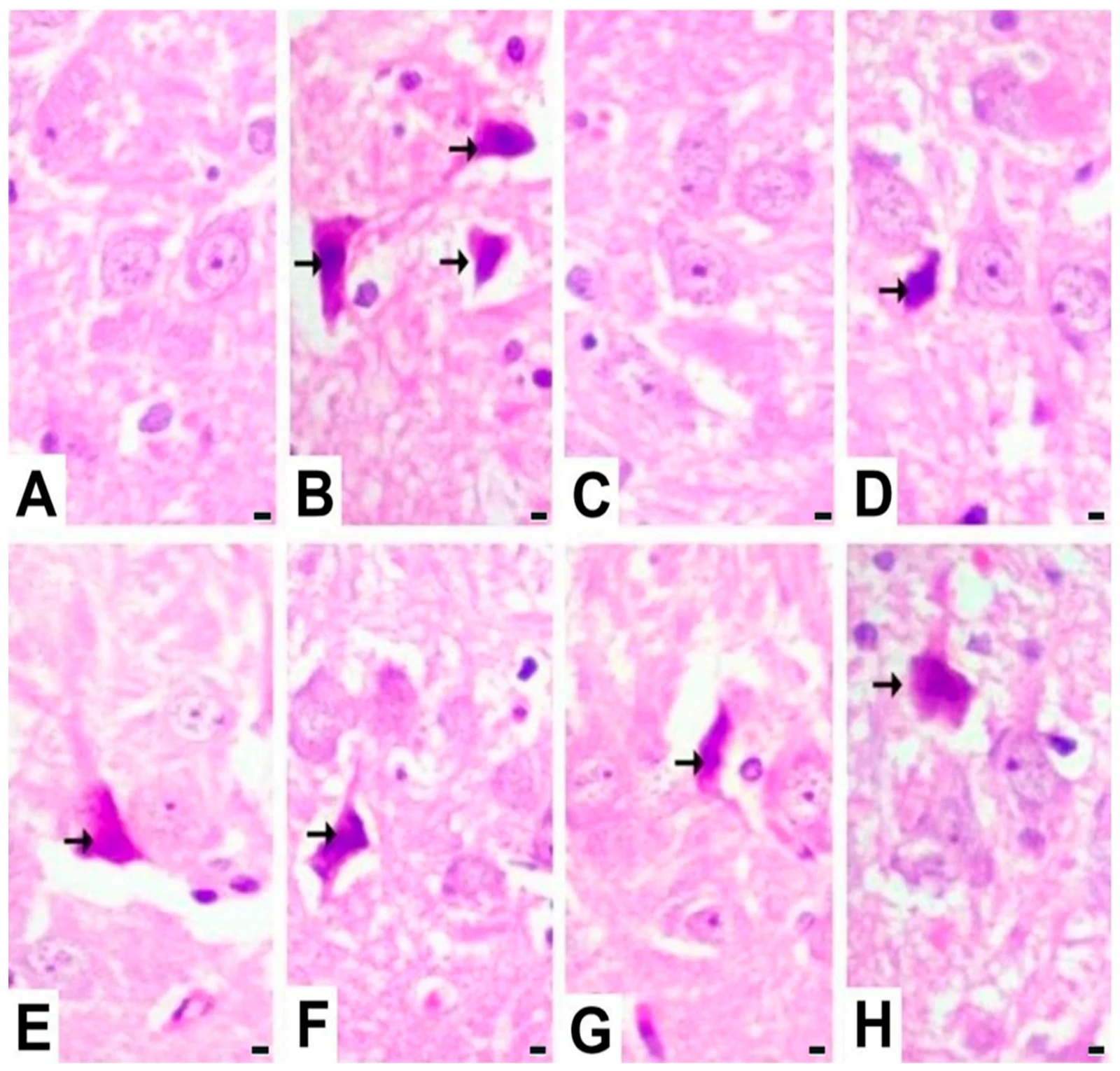
Histopathological changes in the cornu ammonis CA3 region revealed by hematoxylin and eosin (H&E) staining. (**A**) Control group (G1) showing preserved histological architecture. (**B**) STZ group (G2) showing marked neuronal degeneration. (**C**) Sham group (G3) showing preserved histological architecture without evident neuronal degeneration. (**D**) GA100 (G4), (**E**) GA200 (G5), (**F**) EA25 (G6), (**G**) EA50 (G7), and (**H**) memantine (G8) groups showing mild neuronal degeneration compared with the STZ group. Degenerative neurons (→) are indicated. Scale bar: 2 µm.

**Figure 6:**
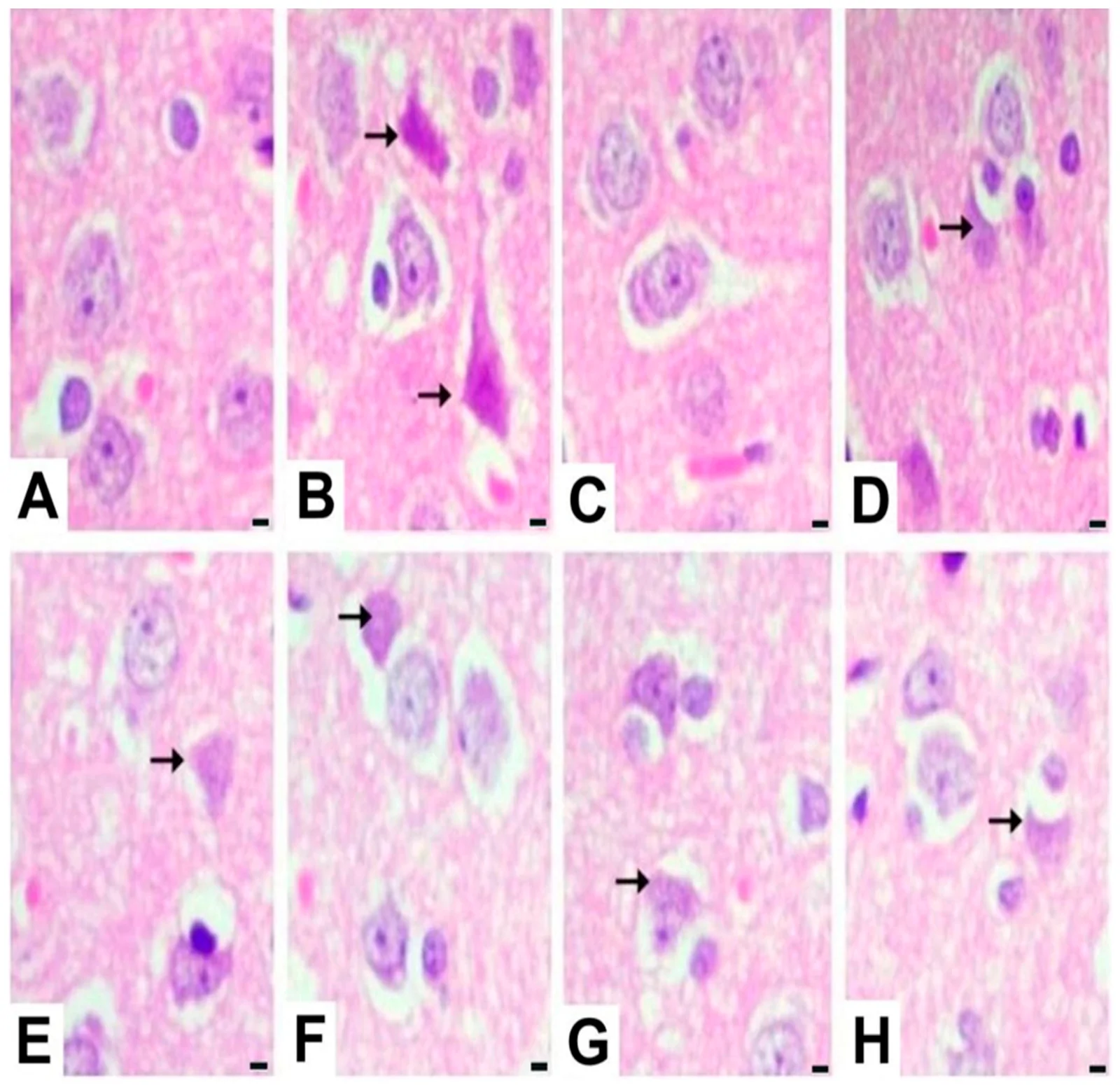
Histopathological changes in the cortical region revealed by hematoxylin and eosin (H&E) staining. (**A**) Control group (G1) showing preserved histological architecture. (**B**) STZ group (G2) showing marked neuronal degeneration. (**C**) Sham group (G3) showing preserved histological architecture without evident neuronal degeneration. (**D**) GA100 (G4), (**E**) GA200 (G5), (**F**) EA25 (G6), (**G**) EA50 (G7), and (**H**) memantine (G8) groups showing mild neuronal degeneration compared with the STZ group. Degenerative neurons (→) are indicated. Scale bar: 2 µm.

Microscopically, the degenerative neurons were characterized by cellular shrinkage and hyperchromatic nuclei (Figure 4, Figure 5 and Figure 6).

### 2.4. Immunohistochemical Findings

Immunohistochemical staining was performed to evaluate tau, β-amyloid, and acetylcholinesterase (AChE) immunoreactivity in the hippocampal CA1/CA2 and CA3 subregions and cerebral cortex. To facilitate group-level comparison of the overall Alzheimer-like molecular burden, an overall mean immunopositivity score was calculated by averaging the semiquantitative scores obtained for tau, β-amyloid, and AChE within each examined region. Marker-specific staining patterns are presented in the corresponding representative figures. Significant differences were observed among the experimental groups (*p* < 0.05; Table 3).

**Figure 7:**
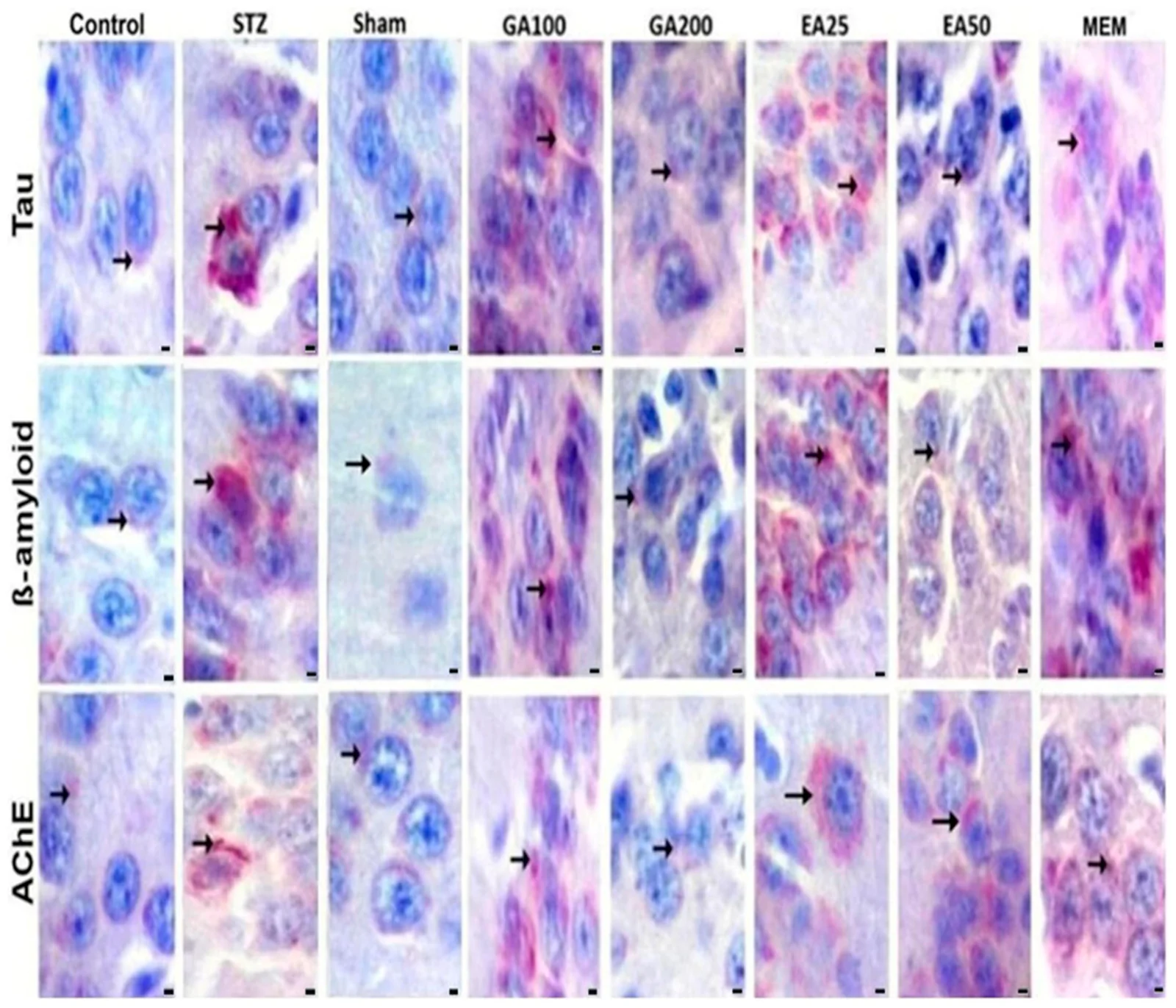
Immunohistochemical staining of tau, β-amyloid, and AChE in the CA1/CA2 region. The control and sham groups exhibited weak immunopositivity. The STZ group showed strong immunopositivity, whereas the GA100, EA25, and memantine groups demonstrated moderate immunopositivity. The GA200 and EA50 groups showed weak immunopositivity. Immunopositive cells are indicated by arrows (→). Scale bar: 2 µm.

**Figure 8:**
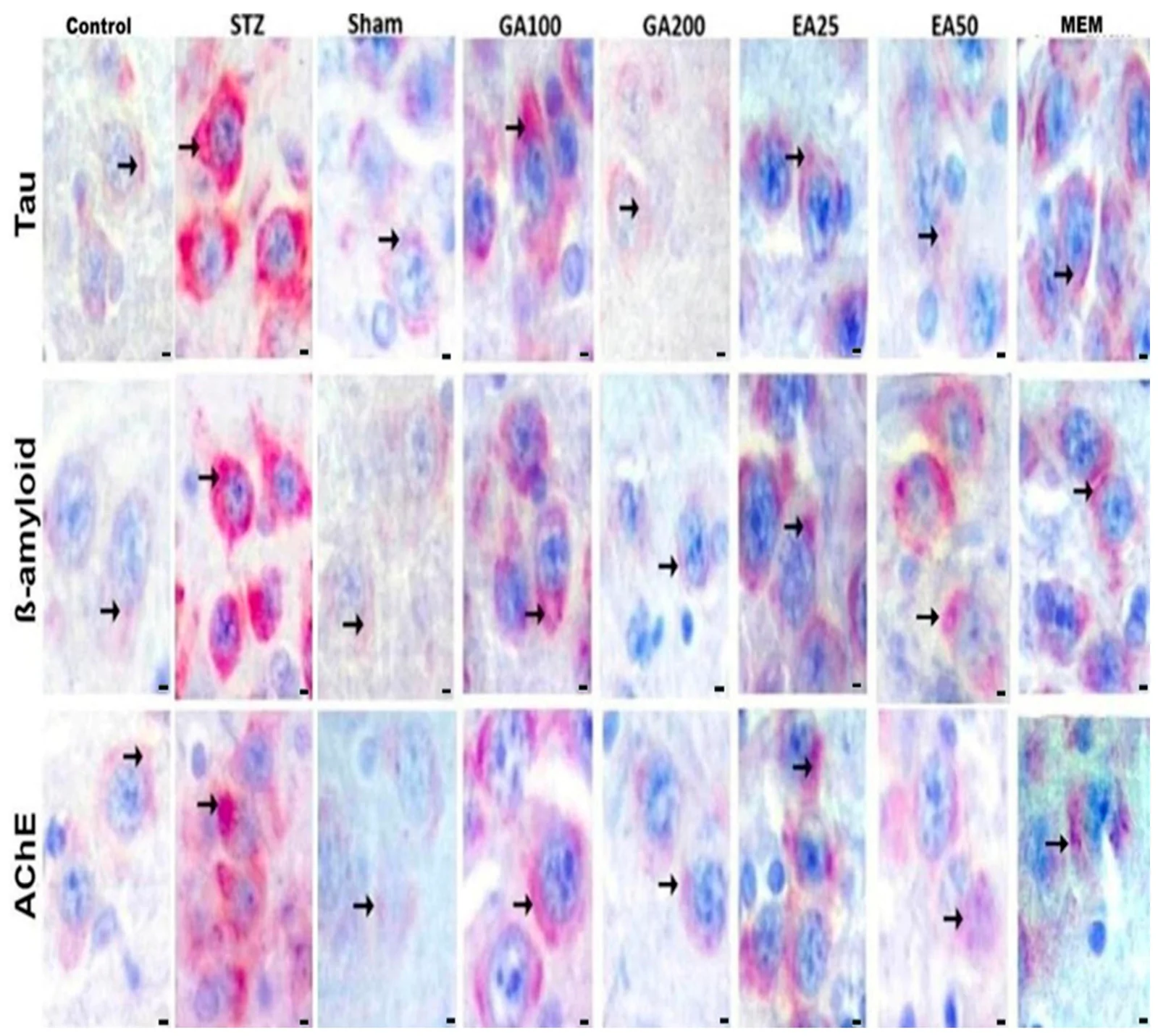
Immunohistochemical staining of tau, β-amyloid, and AChE in the CA3 region. The control and sham groups exhibited weak immunopositivity. The STZ group showed strong immunopositivity, whereas the GA100, EA25, and memantine groups demonstrated moderate immunopositivity. The GA200 and EA50 groups showed weak immunopositivity. Immunopositive cells are indicated by arrows (→). Scale bar: 2 µm.

**Figure 9:**
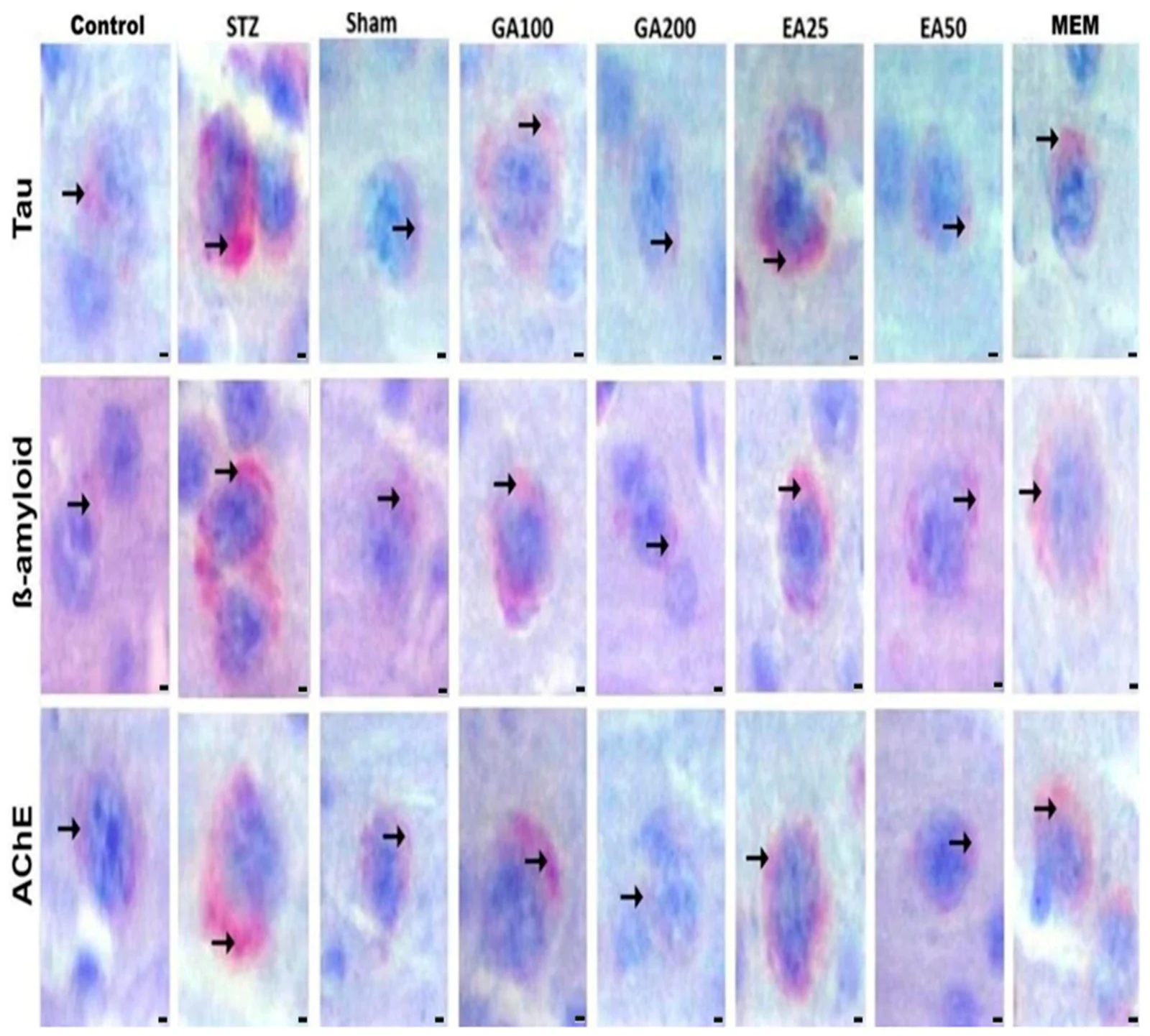
Immunohistochemical staining of tau, β-amyloid, and AChE in the cortical region. The control and sham groups exhibited weak immunopositivity. The STZ group showed strong immunopositivity, whereas the GA100, EA25, and memantine groups demonstrated moderate immunopositivity. The GA200 and EA50 groups showed weak immunopositivity. Immunopositive cells are indicated by arrows (→). Scale bar: 2 µm.

The STZ group exhibited the highest overall immunopositivity across all examined regions. GA 100 mg/kg, EA 25 mg/kg, and memantine groups showed moderate attenuation, with scores remaining statistically comparable to each other but significantly lower than those of the STZ group. In contrast, GA 200 mg/kg and EA 50 mg/kg produced a more pronounced reduction in overall immunopositivity than their corresponding lower-dose groups, with values approaching those observed in the control and sham groups. No significant differences were observed among the hippocampal CA1/CA2, CA3, and cortical regions within any experimental group. These findings are illustrated in Figure 7, Figure 8 and Figure 9.

### 2.5. In Situ Hybridization Findings

In situ hybridization was performed to evaluate tau, β-amyloid/APP-related, and acetylcholinesterase (AChE) mRNA signals in the hippocampal CA1/CA2 and CA3 subregions and cerebral cortex. To facilitate group-level comparison of the overall transcript-related Alzheimer-like molecular signal, an overall mean hybridization score was calculated by averaging the semiquantitative scores obtained for tau, β-amyloid/APP-related, and AChE-associated signals within each examined region. Marker-specific hybridization patterns are presented in the corresponding representative figures. Significant differences were observed among the experimental groups (*p* < 0.05; Table 4).

**Figure 10:**
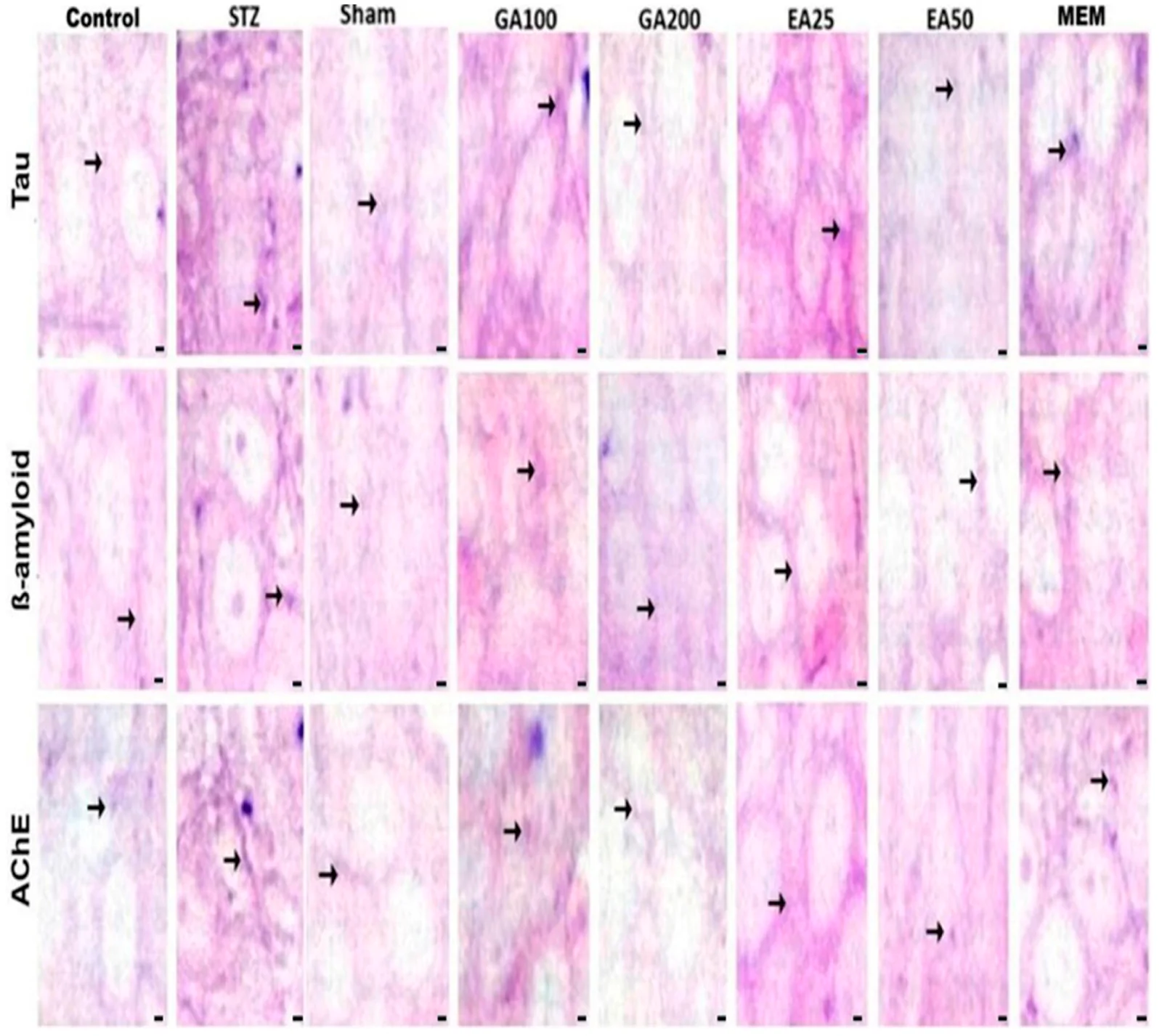
In situ hybridization analysis of tau, β-amyloid/APP-related, and AChE mRNA signals in the CA1/CA2 region. The control and sham groups exhibited low hybridization signals. The STZ group had high hybridization signals, whereas the GA100, EA25, and memantine groups had moderate signals. The GA200 and EA50 groups had low signals. Positive signals are indicated by arrows (→). Scale bar: 2 µm.

**Figure 11:**
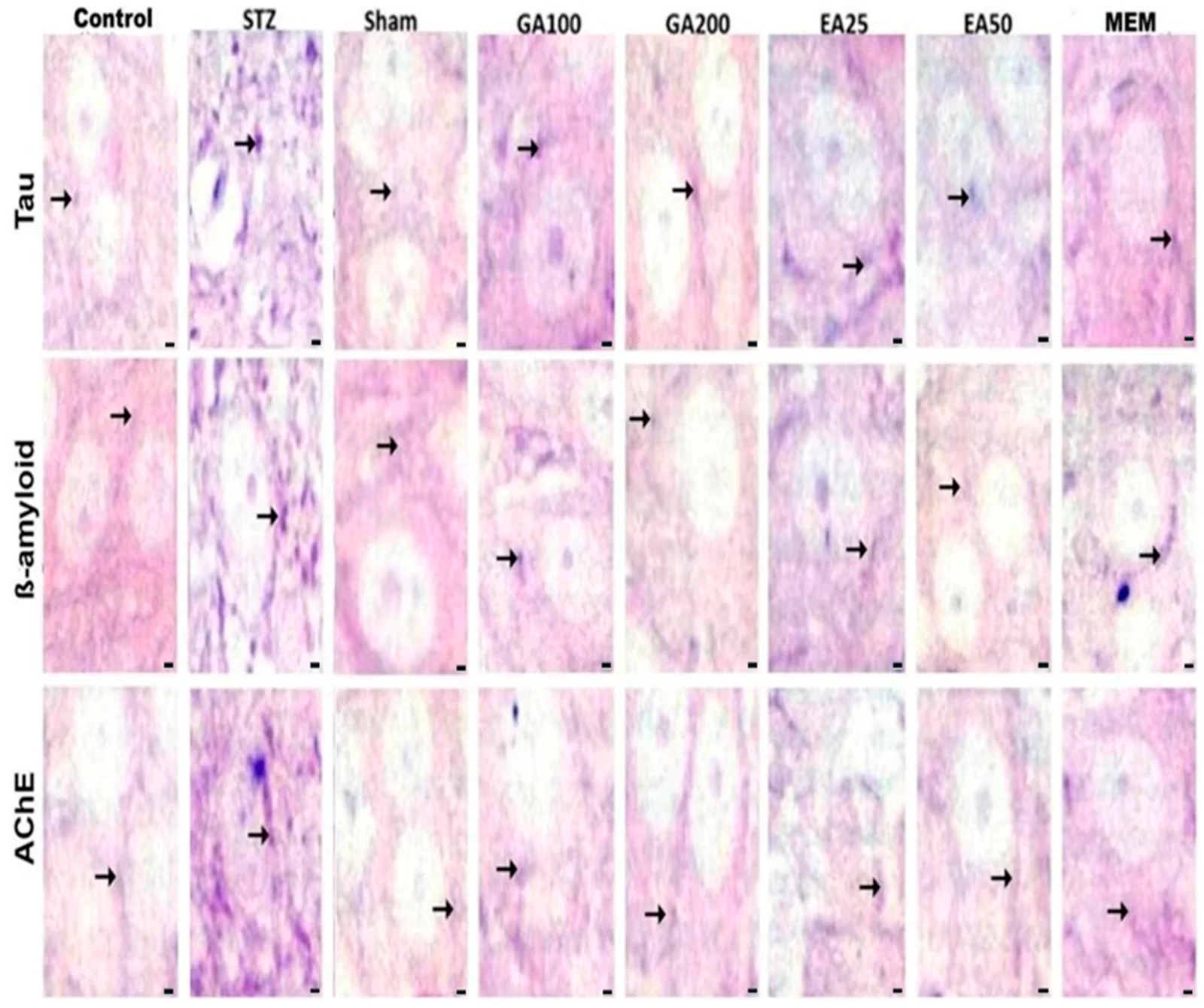
In situ hybridization analysis of tau, β-amyloid/APP-related, and acetylcholinesterase (AChE) mRNA signals in the CA3 region. The control and sham groups exhibited weak hybridization signals. The STZ group showed strong hybridization signals, whereas the GA100, EA25, and memantine groups showed moderate signals. The GA200 and EA50 groups showed weak signals. Positive signals are indicated by arrows (→). Scale bar: 2 µm.

**Figure 12:**
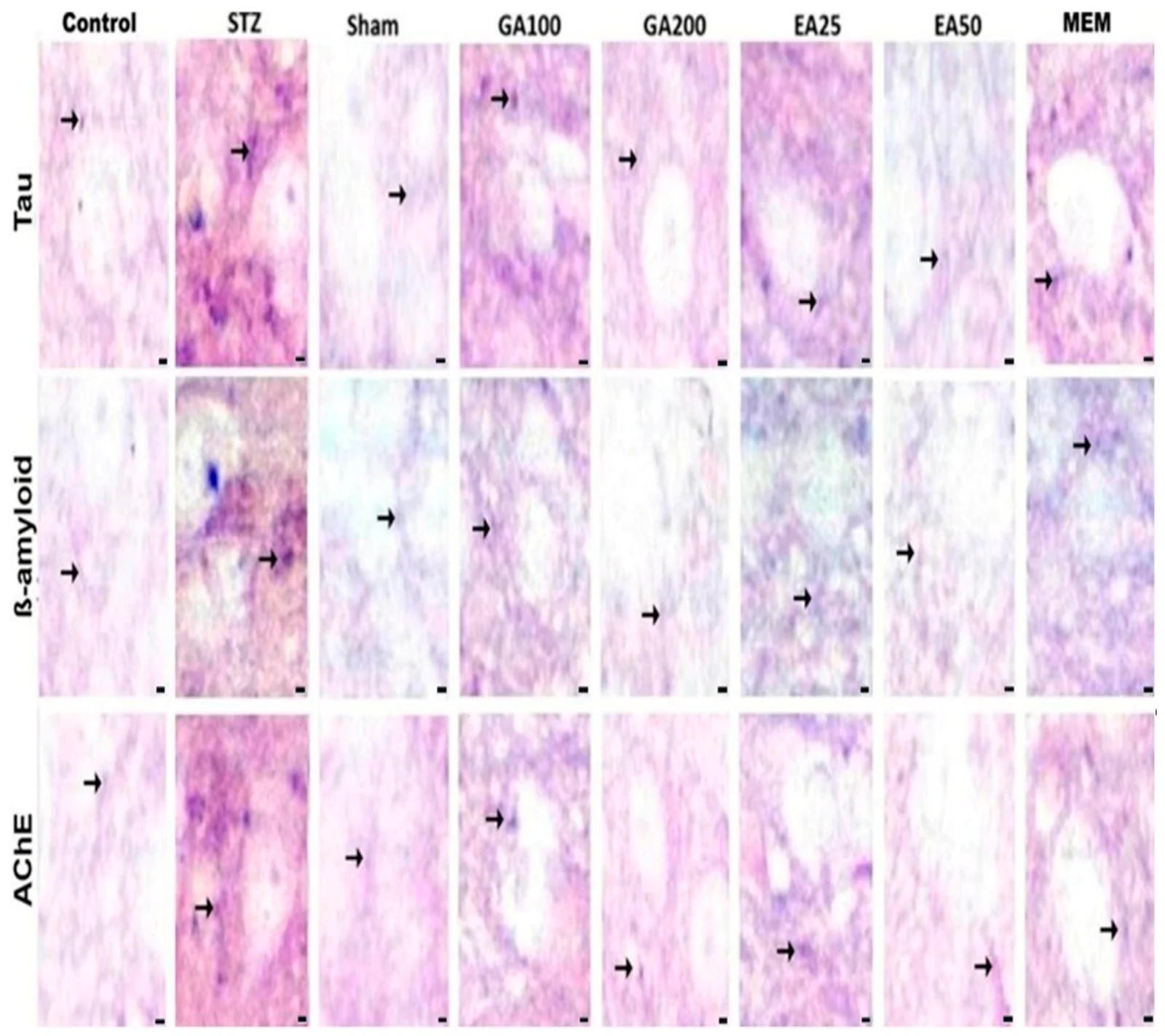
In situ hybridization analysis of tau, β-amyloid/APP-related, and acetylcholinesterase (AChE) mRNA signals in the cortical region. The control and sham groups exhibited weak hybridization signals. The STZ group showed strong hybridization signals, whereas the GA100, EA25, and memantine groups showed moderate signals. The GA200 and EA50 groups showed weak signals. Positive signals are indicated by arrows (→). Scale bar: 2 µm.

The STZ group exhibited the highest overall hybridization scores across all examined regions. GA 100 mg/kg, EA 25 mg/kg, and memantine groups showed moderate attenuation, with scores remaining statistically comparable to each other but lower than those of the STZ group. In contrast, GA 200 mg/kg and EA 50 mg/kg produced a more pronounced reduction in overall hybridization scores than their corresponding lower-dose groups, with values approaching those observed in the control and sham groups. No significant differences were observed among the hippocampal CA1/CA2, CA3, and cortical regions within any experimental group. These findings are illustrated in Figure 10, Figure 11 and Figure 12.

## 3. Discussion

The present study investigated the effects of early administration of gentisic acid and erucic acid in an icv-STZ-induced rat model of sporadic Alzheimer-like pathology using a comprehensive multi-endpoint approach. Because treatment was initiated immediately after STZ administration, before documented cognitive deficits or molecular pathology had developed, the findings should be interpreted as evidence of early neuroprotective/preventive effects rather than reversal of established Alzheimer-like pathology. Under the present experimental conditions, both compounds attenuated the development of STZ-associated behavioral, histopathological, and molecular alterations and were associated with lower total hippocampal tissue glutamate concentrations. In parallel with the behavioral and histopathological findings, GA and EA reduced overall tau, β-amyloid, and AChE immunopositivity, together with lower tau-, β-amyloid/APP-related, and AChE-associated in situ hybridization signals, with the higher doses generally producing more pronounced effects across most endpoints. Among the tested treatments, EA 50 mg/kg/day was associated with the greatest reduction in total hippocampal tissue glutamate concentrations relative to the STZ group, whereas GA 200 mg/kg/day and EA 50 mg/kg/day produced several histological and molecular marker profiles approaching those observed in the control and sham groups. STZ-associated behavioral impairment was evidenced by shorter step-through latency in the passive avoidance test and prolonged escape latency in the Morris water maze, consistent with the established behavioral profile of the icv-STZ model [21,23]. Both GA and EA were associated with relative preservation of behavioral performance, although the magnitude of the observed effects varied across tests and doses. The most consistent preservation of behavioral performance was observed with GA 200 mg/kg/day and EA 50 mg/kg/day, which produced latency values approaching those of the control group in several comparisons. The GA-related findings are broadly consistent with a recent study reporting improved cognitive performance and attenuation of oxidative, inflammatory, cholinergic, amyloid-β, and tau-related alterations in another experimental Alzheimer-like model [26]. However, the present study evaluated GA and EA in the icv-STZ model and examined their associations with total hippocampal glutamate concentrations together with behavioral, histopathological, immunohistochemical, and in situ hybridization endpoints. The heatmaps from the Morris water maze probe trial further supported the behavioral findings, showing greater occupancy of the target quadrant in the higher-dose treatment groups than in the STZ group. Notably, GA 100 mg/kg/day did not produce a statistically significant improvement in the passive avoidance test on either testing day, suggesting that its behavioral efficacy may be dose- and endpoint-dependent. The passive avoidance findings should be interpreted cautiously. The icv-STZ group showed shorter step-through latencies on both post-treatment assessment days, whereas GA200 and EA50 showed the most consistent preservation of conditioned avoidance behavior. However, the ceiling effect observed in the control group limited the sensitivity of the test to detect between-session changes. Furthermore, because locomotor activity was not independently assessed, a potential contribution of motor performance to step-through latency cannot be excluded. Accordingly, these findings support relative preservation of conditioned avoidance behavior but should not be interpreted as definitive evidence of enhanced memory performance.

Recent studies provide additional context for the present findings. Niño-Rivero et al. reported that astrogliosis preceded recognition-memory impairment and cholinergic alterations during the temporal progression of the icv-STZ model [33]. Similarly, Dunacka et al. showed that central IGF-1 treatment improved working and reference memory while reducing neuroinflammation and amyloid-β deposition in this model [34]. A recent systematic review of caffeic acid also supports the multitarget neuroprotective potential of phenolic acids across oxidative, inflammatory, cholinergic, amyloid-β, and tau-related pathways [35]. Collectively, these findings support the relevance of a multi-endpoint approach, without implying that GA or EA acts through identical mechanisms.

This pattern may indicate that the lower dose of GA was insufficient to fully counteract STZ-associated cognitive impairment under the present experimental conditions. Given the reported antioxidant and anti-inflammatory properties of GA, stronger effects at the higher dose may reflect broader engagement of cytoprotective pathways; however, this interpretation remains indirect because oxidative and inflammatory markers were not directly assessed in the present study. Previous evidence supports the neuroprotective effects of GA in experimental Alzheimer-like pathology [26]. However, the endpoint-dependent pattern observed in the present study suggests that its behavioral effects may vary according to dose and assessment method, although the study was not designed to establish a dose–response mechanism. LC–MS/MS analysis showed higher total hippocampal glutamate concentrations in the STZ group than in the control group. Compared with the STZ group, the treatment groups showed lower total hippocampal glutamate concentrations, with EA 50 mg/kg/day producing the largest reduction. These findings indicate treatment-associated differences in total tissue glutamate under the present experimental conditions. However, because glutamate was measured in hippocampal tissue homogenates, the results do not distinguish extracellular glutamate from intracellular or metabolic pools and cannot directly demonstrate impaired glutamate clearance or glutamate-mediated excitotoxicity. Further studies using extracellular glutamate measurements and glutamate-transporter analyses are required to clarify the underlying mechanisms. The memantine group also showed lower total hippocampal glutamate concentrations (79.32 µM) than the STZ group. Although memantine is an NMDA receptor antagonist [18], this tissue-homogenate measure cannot establish a direct relationship with NMDA receptor activity or extracellular glutamate signaling. Interestingly, GA 100 mg/kg/day produced a greater reduction in total hippocampal glutamate concentrations than GA 200 mg/kg/day, whereas the higher dose showed more evident improvements in selected behavioral and histopathological endpoints. This discordance indicates that total tissue glutamate concentrations alone do not explain all endpoint patterns observed with GA. Previous studies suggest that GA may act through antioxidant and anti-inflammatory pathways [25,26]; however, these mechanisms were not directly assessed in the present study. Further dose-ranging and mechanistic studies are required to clarify the basis of this non-linear pattern. Histopathological evaluation revealed marked neuronal degeneration in the STZ group across the hippocampal CA1/CA2, CA3, and cortical regions, with the CA3 subfield showing the most pronounced changes. This regional pattern is consistent with the reported vulnerability of hippocampal neurons to metabolic and excitotoxic insult in experimental neurodegeneration models [14,23]. Compared with the STZ group, the treatment groups showed reduced degeneration scores, with GA 200 mg/kg/day and EA 50 mg/kg/day producing values approaching those observed in the control and sham groups. These histological findings support the behavioral results and suggest that the cognitive improvements observed in the higher-dose groups were accompanied by attenuation of structural neuronal injury. The neuroprotective profile of GA is broadly consistent with its reported antioxidant and cytoprotective effects in neurotoxicity models, whereas the effects of EA may be compatible with mechanisms involving lipid-mediated and PPAR-δ-related neuroprotective pathways; however, the latter mechanism was not directly tested in this study [27,31]. Immunohistochemical analysis showed increased overall tau, β-amyloid, and AChE immunopositivity in the STZ group across the examined regions. GA 200 mg/kg/day and EA 50 mg/kg/day produced a more pronounced reduction in overall immunopositivity than their corresponding lower-dose groups, with values approaching those observed in the control and sham groups. In contrast, GA 100 mg/kg/day, EA 25 mg/kg/day, and memantine showed moderate attenuation. These findings suggest that GA and EA may influence multiple Alzheimer-like molecular markers under the present experimental conditions. However, because the immunohistochemical results were evaluated semiquantitatively and as overall mean immunopositivity scores, marker-specific quantitative confirmation by Western blotting, ELISA, or image-based densitometric analysis would strengthen this interpretation. The reduction in β-amyloid immunopositivity following GA treatment may be consistent with the known relationship between oxidative stress, inflammation, and amyloidogenic processes [5,24]. Similarly, the attenuation of tau immunopositivity may reflect downstream effects associated with reduced cellular stress and neuronal injury rather than a direct effect on tau phosphorylation pathways, which were not specifically assessed in this study. The decrease in AChE immunopositivity also suggests a potential cholinergic component of the observed neuroprotective profile. Nevertheless, direct conclusions regarding cholinergic neurotransmission cannot be made without functional measurements of acetylcholine levels, AChE enzymatic activity, or cholinergic receptor signaling.

In situ hybridization findings generally paralleled the immunohistochemical results, showing higher overall tau-, β-amyloid/APP-related, and AChE-associated hybridization signals in the STZ group and lower signals in the higher-dose GA and EA groups. This concordance suggests that the protein-level immunopositivity changes were accompanied by parallel alterations in transcript-related ISH signals. However, these findings should not be interpreted as definitive evidence of transcriptional regulation, because the ISH analysis was semiquantitative and pathway-specific gene regulation was not directly validated by quantitative PCR or mechanistic assays. Therefore, the observed ISH pattern should be considered supportive rather than conclusive evidence of molecular modulation.

Across the assessed endpoints, GA 200 mg/kg/day and EA 50 mg/kg/day generally produced stronger effects than the corresponding lower doses. However, the non-linear glutamate response observed with GA indicates that the overall pharmacological profile was endpoint-dependent rather than uniformly dose-dependent. EA 50 mg/kg/day showed the most prominent association with lower total hippocampal tissue glutamate concentrations and attenuated Alzheimer-like histological and molecular alterations. Under the present experimental conditions, EA 50 mg/kg/day appeared to show a broader endpoint-associated profile than memantine across glutamate, histological, and molecular readouts. However, this observation should not be interpreted as pharmacological superiority, because the study was not designed as a direct comparative efficacy trial. Future studies should assess PPAR-δ signaling, glutamate transporter expression, NMDA receptor subunits, oxidative stress, inflammation, and cholinergic enzymatic activity to clarify the pathways underlying these effects.

### Limitations and Future Perspectives

Several limitations warrant explicit acknowledgment. First, while total hippocampal tissue glutamate was quantified using LC–MS/MS, the study did not directly assess EAAT expression, vesicular glutamate transporter (VGLUT) activity, or NMDA receptor subunit composition. The mechanisms underlying the observed treatment-associated differences in total hippocampal tissue glutamate remain unclear because the present analysis did not distinguish extracellular glutamate from intracellular or metabolic pools. Second, the proposed involvement of PPAR-δ signaling in mediating the effects of EA was not pharmacologically or genetically confirmed; future studies employing selective PPAR-δ antagonists or pathway-specific gene expression analyses are needed to validate this hypothesis. Third, the icv-STZ model does not fully reproduce the progressive amyloid deposition, tau spreading, and long-term neurodegenerative trajectory of human AD, and extrapolation to clinical settings should be tempered accordingly. Fourth, this study included endpoint measurements only; therefore, longitudinal studies are needed to determine the temporal relationship between changes in total hippocampal tissue glutamate and the observed behavioral, structural, and molecular alterations. Fifth, the semiquantitative and composite nature of the histopathological, immunohistochemical, and in situ hybridization assessments limits marker-specific interpretation. Quantitative analyses of individual molecular markers using complementary methods, such as Western blotting, ELISA, or image-based densitometry, would strengthen the molecular characterization of the observed effects. Sixth, no dedicated toxicological assessment, clinical chemistry analysis, or peripheral organ histopathology was performed; therefore, the present findings do not establish the systemic safety of GA or EA at the administered doses. Seventh, this study was conducted exclusively in female rats. Although this choice was motivated by the higher epidemiological burden of AD in women and reported sex-related differences in hippocampal glutamatergic and cholinergic function, the findings cannot be directly extrapolated to male animals or mixed-sex populations. Future studies including both sexes are required to determine whether the neuroprotective effects of GA and EA are sex-dependent. Despite these limitations, the observed differences in total hippocampal tissue glutamate occurred alongside behavioral, histopathological, and molecular changes; however, the present findings do not establish a functional or causal relationship between these endpoints.

## 4. Materials and Methods

All the experimental procedures were conducted at the Neuroscience and Behavioral Laboratories of the Department of Medical Pharmacology, Atatürk University Faculty of Medicine (Erzurum, Türkiye).

Induction of the icv-STZ model, behavioral assessments, postoperative care, animal monitoring, euthanasia, and brain tissue collection were performed at this facility.

Histopathological, immunohistochemical, and in situ hybridization analyses were carried out at the Department of Pathology, Cumhuriyet University Faculty of Medicine.

LC–MS/MS analyses were performed at the Eastern Anatolia High Technology Application and Research Center (DAYTAM), Atatürk University.

### 4.1. Chemicals and Reagents

Erucic acid (cis-13-docosenoic acid, 90% (CS), product code: 102215950) and gentisic acid (2,5-dihydroxybenzoic acid, 98%, CAS No. 490-79-9) were purchased from Sigma-Aldrich (St. Louis, MO, USA). Streptozotocin (STZ; ≥98% (HPLC), product No. S0130-100MG) was also obtained from Sigma-Aldrich. Memantine hydrochloride was used in tablet form (Demax®, Abdi İbrahim, Istanbul, Türkiye). Primary antibodies against acetylcholinesterase (AChE), tau, and β-amyloid were obtained from Santa Cruz Biotechnology (Dallas, TX, USA) and used for immunohistochemical analyses. In situ hybridization reagents (IsHyb™, AMSBIO, Abingdon, UK) and proteinase K (Sigma-Aldrich) were used according to the manufacturers’ instructions. Ketamine (Dutch Farm International B.V., Nederhorst den Berg, The Netherlands) and xylazine (Bioveta, Ivanovice na Hané, Czech Republic) were used for anesthesia.

### 4.2. Experimental Animals

A total of 64 adult female Sprague–Dawley rats (270–310 g) were used in this study. The animals were housed under controlled environmental conditions (22 ± 2 °C, 65–70% humidity) with a 12 h light/12 h dark cycle and ad libitum access to standard laboratory chow and water. All experimental procedures were approved by the Atatürk University Animal Experiments Local Ethics Committee (Approval No: E-42190979-000-2100152485; Date: 14 June 2021) and conducted in accordance with international guidelines for the care and use of laboratory animals. The study design, conduct, and reporting complied with the relevant guidelines to ensure methodological rigor and reproducibility.

### 4.3. Experimental Design and Group Allocation

A total of 64 female Sprague–Dawley rats were randomly assigned to eight experimental groups (*n* = 8 per group) using a computer-generated randomization method. The sample size of eight animals per group was determined a priori on the basis of a power analysis (power = 0.82, α = 0.05) using data from previously published icv-STZ studies and was considered sufficient to detect biologically relevant differences across groups [23,36].

The doses of all pharmacological agents were selected on the basis of previously published in vivo studies reporting biological activity at these dose ranges in rodent models. Streptozotocin was administered at 3 mg/kg bilaterally via the intracerebroventricular route, a dose consistently reported to induce reliable cognitive impairment and AD-like neuropathology without causing acute mortality [21,23]. Gentisic acid was administered orally at dosages of 100 and 200 mg/kg/day on the basis of doses previously shown to exert neuroprotective and anti-inflammatory effects in rodent neurotoxicity models [25,26]. Erucic acid was administered orally at 25 and 50 mg/kg/day in accordance with doses reported to produce memory-enhancing and neuroprotective effects in rodents [29,31]. Memantine was orally administered at a dosage of 10 mg/kg/day, which is widely used as a positive control in icv-STZ-induced AD rat models and corresponds to the therapeutically relevant range in rodents [36,37]. Gentisic acid and memantine were prepared in distilled water, whereas erucic acid was prepared in corn oil because of its lipophilic nature. Memantine tablets were finely powdered and suspended in distilled water immediately before oral administration. All preparations were freshly prepared before administration and administered orally by gavage once daily for 21 consecutive days. The oral gavage volume was standardized to 10 mL/kg body weight for all treatment groups.

The experimental groups were defined as follows:

- Control group: no intervention;
- STZ group: intracerebroventricular (i.c.v.) administration of streptozotocin (3 mg/kg);
- Sham group: i.c.v. administration of citrate buffer (vehicle control);
- STZ + GA (100 mg/kg/day): oral GA was administered for 21 consecutive days following STZ injection;
- STZ + GA (200 mg/kg/day): oral GA was administered for 21 consecutive days following STZ injection;
- STZ + EA (25 mg/kg/day): oral EA was administered for 21 consecutive days following STZ injection;
- STZ + EA (50 mg/kg/day): oral EA was administered for 21 consecutive days following STZ injection;
- STZ + MEM (10 mg/kg/day): oral MEM was administered for 21 consecutive days following STZ injection.

All treatments were initiated immediately after STZ administration and continued once daily for 21 consecutive days. The 21-day treatment duration was selected on the basis of evidence that this period is sufficient for the establishment of AD-like neuropathological changes and the detection of pharmacological effects in the icv-STZ-induced rat model [23,36]. Behavioral assessments were performed after the treatment period was completed. The animals were subsequently euthanized under anesthesia, and brain tissues were collected for further biochemical, molecular, and histopathological analyses. All experimental procedures, including behavioral testing and tissue analyses, were conducted by investigators who were blinded to group allocation.

#### 4.3.1. Timeline of Treatment and Tissue Collection

All pharmacological treatments were initiated immediately following intracerebroventricular (i.c.v.) administration of STZ and were continued once daily for 21 consecutive days. At the end of the treatment period, behavioral assessments were performed. The animals were subsequently euthanized under sevoflurane anesthesia, after which the brain tissues were rapidly harvested for biochemical, molecular, and histopathological analyses.

#### 4.3.2. Induction of the icv-STZ Model

An experimental model of sporadic Alzheimer-like pathology was induced by i.c.v. administration of STZ.

The animals were anesthetized with ketamine/xylazine and secured in a stereotaxic apparatus. On the basis of a rat brain atlas, bilateral lateral ventricles were targeted using the following coordinates relative to the bregma: 0.8 mm posterior, 1.5 mm lateral, and 3.6 mm deep.

Under aseptic conditions, burr holes were drilled at the appropriate stereotaxic coordinates. STZ was freshly prepared in cold citrate buffer (pH 4.4) immediately before injection and administered bilaterally into the lateral ventricles at a total dose of 3 mg/kg using a 10-µL Hamilton microsyringe. A total injection volume of 10 µL was divided equally between the two ventricles, with 5 µL injected into each ventricle over 1 min. After each injection, the needle was left in place for 2 min to minimize reflux along the injection tract and then slowly withdrawn. Sham-operated animals received the same total volume of citrate buffer (pH 4.4), divided equally between the two lateral ventricles, using the same stereotaxic procedure. Following injection, the incision site was sutured, and the animals were allowed to recover under standard laboratory conditions. All behavioral tests, LC–MS/MS analyses, in situ hybridization, histopathological evaluation, and immunohistochemical assessments were performed by independent investigators blinded to group allocation.

### 4.4. Behavioral Assessments

#### 4.4.1. Morris Water Maze Test

Spatial learning and memory were assessed using the Morris water maze (MWM), a well-established paradigm for evaluating hippocampus-dependent cognitive function. The apparatus consisted of a circular pool (150 cm in diameter, 45 cm in height) filled with water rendered opaque using nontoxic white tempera paint and maintained at 24 ± 1 °C. The pool was virtually divided into four equal quadrants (north, south, east, and west). A square escape platform (12 × 12 cm) was submerged 3 cm below the water surface and positioned in the center of the target quadrant. The platform location remained constant throughout the acquisition phase. Prominent distal visual cues were placed around the testing room and remained unchanged during the experiment.

##### Acquisition Phase

The animals were trained for four consecutive days, with one trial per day, conducted at a fixed time (09:00 a.m.) to minimize circadian variability. In each trial, the rats were released into the pool from semirandomized starting points (north, south, east, or west), ensuring a balanced distribution across days. Each rat was allowed a maximum of 90 s to locate the hidden platform. Animals that failed to find the platform within this period were gently guided to it and allowed to remain on the platform for 15 s to facilitate spatial learning. Escape latency, swim distance, and swimming speed were recorded as primary indices of learning performance.

##### Probe Trial

On day 5, the platform was removed, and the animals were allowed to swim freely for 60 s. The time spent in the target quadrant, number of crossings over the former platform location, and search strategies were analyzed as indices of spatial memory retention.

##### Data Acquisition and Blinding

All trials were recorded using a ceiling-mounted video tracking system and analyzed using automated tracking software to minimize observer bias. Behavioral testing and data analysis were performed by investigators who were blinded to group allocation. The experimental design, conduct, and reporting adhered to the guidelines.

#### 4.4.2. Passive Avoidance Test

Learning and memory performance were evaluated using the passive avoidance test, a well-established paradigm for assessing associative learning and memory retention. The test was performed using a two-compartment step-through passive avoidance apparatus (MAY passive avoidance cage; Commat Ltd., Ankara, Türkiye). The apparatus consisted of two equal-sized compartments, a brightly illuminated chamber and a dark chamber, separated by an automated guillotine door. The floor of both compartments was composed of stainless-steel rods connected to an electrical shock generator. All the experiments were conducted under controlled environmental and lighting conditions.

##### Pre-Intervention Conditioning Session

Before icv-STZ or vehicle administration and the initiation of pharmacological treatments, all animals underwent a passive avoidance conditioning session. Each rat was placed in the illuminated compartment, and after a 20-s habituation period, the guillotine door was opened to provide access to the dark compartment. Upon full entry into the dark compartment, the door was closed automatically, and a mild foot shock (0.5 mA for 3 s) was delivered through the grid floor. Thus, all animals received the conditioning stimulus before the experimental interventions.

##### Post-Treatment Assessment Sessions

Following completion of the 21-day treatment period, passive avoidance performance was assessed on two consecutive days, designated post-treatment Day 1 and Day 2. During each assessment session, each rat was placed in the illuminated compartment, and the latency to enter the dark compartment was recorded, with a cut-off time of 300 s. Upon full entry into the dark compartment, the door was closed automatically, and a foot shock (0.5 mA for 3 s) was delivered through the grid floor. Animals that did not enter the dark compartment within 300 s were assigned the cut-off value and did not receive a foot shock during that assessment session. Because all animals had undergone preconditioning before the experimental interventions, failure to enter the dark compartment during a post-treatment assessment did not indicate an absence of prior conditioning.

##### Data Acquisition and Blinding

Behavioral responses were recorded and analyzed under standardized conditions. All behavioral assessments and data analyses were performed by investigators who were blinded to group allocation.

### 4.5. LC–MS/MS Analysis of Glutamate

Total glutamate concentrations in hippocampal tissue homogenates were quantified using liquid chromatography–tandem mass spectrometry (LC–MS/MS). Approximately 200 mg of hippocampal tissue was homogenized in 1 mL of ice-cold acetonitrile using a TissueLyser II system (QIAGEN, Hilden, Germany) for 2 min with stainless steel beads. The homogenates were incubated at −20 °C for 1 h to ensure efficient protein precipitation. The samples were subsequently centrifuged at 12,000× *g* for 10 min at 4 °C, after which the supernatants were filtered through 0.45 µm membrane filters prior to analysis. Quantification was performed using external calibration curves generated from reference glutamate standards.

Chromatographic separation was performed using an InfinityLab Poroshell 120 SB-AQ column (3.0 × 150 mm, 2.7 µm; Agilent Technologies (Santa Clara, CA, USA)). The mobile phase consisted of 0.1% formic acid in deionized water and acetonitrile (70:30, *v*/*v*) and was delivered at a flow rate of 0.3 mL/min. The column temperature was maintained at 30 °C, and the injection volume was 5 µL. The total run time was 4 min.

Mass spectrometric detection was performed using an Agilent 6460 triple quadrupole LC–MS/MS system (Agilent Technologies, Santa Clara, CA, USA) equipped with an Agilent Jet Stream electrospray ionization source operating in positive-ion mode. Glutamic acid was monitored in multiple reaction monitoring (MRM) mode using the precursor ion at *m*/*z* 147.8 and two product-ion transitions: *m*/*z* 147.8 → 129.9 and *m*/*z* 147.8 → 84.0. The transition *m*/*z* 147.8 → 129.9 was used as the quantifier transition, whereas *m*/*z* 147.8 → 84.0 was used as the qualifier transition. Analyte identification was based on agreement with the retention time of the reference standard (approximately 2.0–2.1 min) and an ion-ratio tolerance of ±20% relative to the reference-standard response. The instrument parameters included a capillary voltage of 4500 V and a nebulizer pressure of 35 psi. Quantification was performed using external calibration curves generated from reference standards. The calibration curves demonstrated excellent linearity (*R*^2^ > 0.99).

Method validation parameters were assessed as follows: The limit of detection (LOD) and limit of quantification (LOQ) were determined on the basis of signal-to-noise ratios (S/N = 3 and 10, respectively). Recovery rates were evaluated by spiking known concentrations of glutamate into tissue samples and were within acceptable ranges (typically 85–115%). Matrix effects were assessed by comparing signal responses in matrix-matched standards versus those in solvent standards. Intra- and interday precision were evaluated and are expressed as the relative standard deviation (RSD), with values < 15% considered acceptable.

### 4.6. Histopathological Analysis

Following sacrifice, the brain tissues were rapidly removed and fixed in 10% neutral-buffered formalin. After fixation, the tissues were processed through a graded series of alcohol and xylene and embedded in paraffin. Serial coronal sections (5 µm thick) were obtained and mounted onto poly-L-lysine-coated slides. Sections were stained with hematoxylin and eosin (H&E) according to standard protocols. Histopathological evaluation of the cerebral cortex and hippocampal regions (CA1, CA2, and CA3) was performed. Neuronal degeneration was assessed on the basis of established morphological criteria, including neuronal shrinkage, cytoplasmic eosinophilia, and nuclear pyknosis.

Degenerative changes were evaluated semiquantitatively using a scoring system: 0 (no damage), 1 (mild), 2 (moderate), and 3 (severe). For each animal, at least 5–10 randomly selected nonoverlapping fields per section were examined under light microscopy (400× magnification), and mean scores were calculated. All evaluations were performed by an experienced pathologist who was blinded to group allocation.

### 4.7. Immunohistochemical Analysis

Paraffin-embedded brain tissues were cut into 4 µm thick sections and mounted onto poly-L-lysine-coated slides. The sections were deparaffinized in xylene and rehydrated through a graded ethanol series, followed by washing in phosphate-buffered saline (PBS). Endogenous peroxidase activity was blocked by incubating the sections with 3% hydrogen peroxide (H_2_O_2_) for 10 min at room temperature. Antigen retrieval was performed using appropriate retrieval buffer (e.g., citrate buffer, pH 6.0) in a microwave oven at 500 W for 2 × 5 min. After cooling to room temperature, the sections were washed with PBS. The sections were incubated overnight at 4 °C with the following primary antibodies: Tau (Santa Cruz Biotechnology, Dallas, TX, USA; sc-390476; 1:2000). β-Amyloid (Santa Cruz, sc-28365; 1:100). Acetylcholinesterase (AChE) (Santa Cruz, sc-373901; 1:100). Following primary antibody incubation, the sections were treated with a horseradish peroxidase (HRP)-conjugated secondary antibody using a commercial detection system (Thermo Fisher Scientific, Waltham, MA, USA; TP-125-HL) according to the manufacturer’s instructions. Immunoreactivity was visualized using 3-amino-9-ethylcarbazole (AEC) as the chromogen. The sections were counterstained with Mayer’s hematoxylin, mounted with aqueous mounting medium, and examined under a light microscope (Olympus BX53, Tokyo, Japan).

#### 4.7.1. Controls and Validation

- Negative controls (primary antibody omitted);
- Positive control tissues (where applicable);
- Antibody specificity was confirmed on the basis of the manufacturer’s validation and the literature.

#### 4.7.2. Semiquantitative Evaluation

Because the primary aim of this analysis was to compare the overall Alzheimer-like molecular burden across experimental groups and brain regions, an overall mean immunopositivity score was calculated by averaging the semiquantitative scores obtained for tau, β-amyloid, and AChE within each region. Marker-specific staining patterns are presented in the corresponding representative figures.

Immunopositivity was evaluated in the cerebral cortex and hippocampal regions (CA1, CA2, and CA3). Staining intensity and distribution were assessed semiquantitatively using a scoring system: 0 (no staining), 1 (weak), 2 (moderate), and 3 (strong).

For each animal, multiple nonoverlapping fields were examined (e.g., at 400× magnification), and mean scores were calculated.

All evaluations were performed by a blinded observer. Negative control sections were processed in parallel by omitting the primary antibody.

### 4.8. In Situ Hybridization Analysis

In situ hybridization (ISH) was performed to evaluate the expression of acetylcholinesterase (AChE), tau, and APP mRNA in the brain tissue sections.

Paraffin-embedded tissue sections (4–5 µm) were incubated at 57 °C for 1 h, followed by deparaffinization in xylene and rehydration through a graded ethanol series. The sections were subsequently washed in phosphate-buffered saline (PBS) and treated with proteinase K to enhance probe penetration.

Prehybridization was performed using prehybridization buffer at 50 °C for 3 h to reduce nonspecific binding. The sections were subsequently incubated overnight at 45 °C with digoxigenin (DIG)-labeled oligonucleotide probes diluted in hybridization buffer.

After hybridization, the sections were subjected to stringent washing steps to remove nonspecifically bound probes, followed by blocking to prevent nonspecific antibody binding. The sections were then incubated with an anti-digoxigenin antibody (1:300) at room temperature for 4 h. After they were incubated, the sections were washed in PBS (3 × 10 min) and alkaline phosphatase buffer (2 × 5 min). Signal detection was performed using an NBT/BCIP chromogenic substrate under controlled conditions until optimal staining intensity was achieved. The sections were rinsed in distilled water, counterstained, and examined under a light microscope.

#### 4.8.1. Semiquantitative Evaluation

Because the primary aim was to assess the overall transcript-related Alzheimer-like molecular signal rather than to compare each marker separately, an overall mean hybridization score was calculated by averaging the semiquantitative scores obtained for tau, β-amyloid/APP-related, and AChE-associated signals within each region. Marker-specific hybridization patterns are shown in the corresponding representative figures.

Hybridization signals were evaluated in the cerebral cortex and hippocampal regions (CA1, CA2, and CA3). Signal intensity was assessed semiquantitatively using a scoring system: 0 (no signal), 1 (weak), 2 (moderate), and 3 (strong). Multiple nonoverlapping fields were analyzed per section, and mean scores were calculated. All analyses were performed by investigators who were blinded to group allocation. Negative control sections (without probe or with sense probe) were processed in parallel to confirm specificity.

#### 4.8.2. Oligonucleotide Probes

The following DIG-labeled oligonucleotide probes were used:

- Ache: 5dig/cacagacacgctggacgag;
- (Ncbi Reference Sequence: Nm_172009.1);
- Tau: 5dig/catcacaagccaggaggtgg;
- (Ncbi Reference Sequence: Nm_017212.2);
- B-Amyloid: 5dig/ggactgaccactcgaccag (Ncbi Reference Sequence: Nm_019288.2).

### 4.9. Statistical Analyses

All statistical analyses were performed using SPSS software version 20.0 (IBM Corp., Armonk, NY, USA). Data distribution was assessed using the Shapiro–Wilk test, and homogeneity of variance was evaluated using Levene’s test. Parametric data are expressed as mean ± standard deviation (SD), whereas nonparametric data are presented as median and interquartile range (IQR), where appropriate.

For the Morris water maze acquisition phase, escape latency was analyzed using a two-way mixed-design repeated-measures ANOVA, with experimental group as the between-subject factor and training day as the within-subject factor. When significant main effects or interactions were detected, post hoc comparisons were performed using Bonferroni-adjusted pairwise comparisons or Tukey’s test, as appropriate. Morris water maze probe trial parameters, passive avoidance data for each testing day, and hippocampal glutamate levels were analyzed using one-way ANOVA followed by Tukey’s post hoc test when parametric assumptions were met.

Histopathological, immunohistochemical, and in situ hybridization scores were considered ordinal/nonparametric data and were analyzed using the Kruskal–Wallis test. Pairwise comparisons were performed using the Mann–Whitney U test with appropriate correction for multiple comparisons. All tests were two-tailed, and *p* values < 0.05 were considered statistically significant. The sample size (*n* = 8 per group) was determined a priori, and all statistical analyses were performed by investigators blinded to group allocation.

## 5. Conclusions

In this early-intervention design, gentisic acid and erucic acid attenuated the development of STZ-associated behavioral, histopathological, and Alzheimer-like molecular alterations in an icv-STZ-induced rat model. Both compounds were associated with preserved behavioral performance, lower neuronal degeneration scores, and reduced tau-, β-amyloid-, APP-, and AChE-related signals, with the higher doses generally producing more pronounced effects. Among the tested treatments, EA 50 mg/kg/day was associated with the greatest reduction in total hippocampal tissue glutamate. However, this measurement does not distinguish extracellular glutamate from intracellular or metabolic pools and therefore does not directly demonstrate glutamate-mediated excitotoxicity. Because treatment was initiated immediately after STZ administration, these findings support early neuroprotective effects rather than therapeutic reversal of established Alzheimer-like pathology.

## Figures and Tables

**Table 1:** Step-through latencies during the passive avoidance assessment sessions on post-treatment Days 1 and 2.

Groups	Post-Treatment Day 1 (s)	Post-Treatment Day 2 (s)	Mean Change (Day 2–Day 1), Descriptive Only (s)
Control (G1)	300.00 ± 0.00	300.00 ± 0.00	0.00
STZ (3 mg/kg, i.c.v.) (G2)	139.83 ± 27.00 ^##^	133.83 ± 9.90 ^#^	−6.00
Sham (citrate buffer, i.c.v.) (G3)	272.80 ± 33.00 *	300.00 ± 0.00 **	+27.20
GA (100 mg/kg/day) (G4)	240.75 ± 40.00	199.50 ± 9.79	−41.25
GA (200 mg/kg/day) (G5)	262.80 ± 30.00 *	300.00 ± 0.00 **	+37.20
EA (25 mg/kg/day) (G6)	258.33 ± 32.00 *	240.50 ± 8.74 *	−17.83
EA (50 mg/kg/day) (G7)	288.33 ± 35.00 *	300.00 ± 0.00 **	+11.67
Memantine (10 mg/kg/day) (G8)	264.50 ± 38.00 *	264.17 ± 9.55 *	−0.33

Data are presented as mean ± SD. Statistically significant differences are indicated as follows: ^#^ *p* < 0.05 and ^##^ *p* < 0.01 versus the control group; * *p* < 0.05 and ** *p* < 0.01 versus the STZ group. GA, gentisic acid; EA, erucic acid; STZ, streptozotocin.

**Table 2:** Semiquantitative evaluation of neuronal degeneration in the hippocampal (CA1/CA2, CA3) and cortical regions.

Groups	CA1/CA2	CA3	Cortex
Control (G1)	0.33 ± 0.51 ^aA^	0.16 ± 0.40 ^aA^	0.33 ± 0.51 ^aA^
STZ (3 mg/kg, i.c.v.) (G2)	2.16 ± 0.40 ^bA^	2.66 ± 0.51 ^bB^	2.00 ± 0.00 ^bA^
Sham (citrate buffer, i.c.v.) (G3)	0.33 ± 0.51 ^aA^	0.16 ± 0.40 ^aA^	0.16 ± 0.40 ^aA^
GA (100 mg/kg/day) (G4)	1.16 ± 0.40 ^cA^	1.16 ± 0.40 ^cA^	1.00 ± 0.00 ^cA^
GA (200 mg/kg/day) (G5)	1.00 ± 0.00 ^cA^	1.00 ± 0.00 ^cA^	1.16 ± 0.40 ^cA^
EA (25 mg/kg/day) (G6)	1.00 ± 0.00 ^cA^	1.16 ± 0.40 ^cA^	0.83 ± 0.40 ^cA^
EA (50 mg/kg/day) (G7)	0.83 ± 0.40 ^cA^	1.00 ± 0.00 ^cA^	1.00 ± 0.00 ^cA^
Memantine (10 mg/kg/day) (G8)	1.16 ± 0.40 ^cA^	1.16 ± 0.40 ^cA^	1.00 ± 0.00 ^cA^

The data are presented as the mean ± SD. Different lowercase letters (a–c) indicate statistically significant differences between groups within the same column (*p* < 0.05). Different uppercase letters (A, B) indicate statistically significant differences between regions within the same group (*p* < 0.05).

**Table 3:** Overall mean semiquantitative immunohistochemical scores for tau, β-amyloid, and acetylcholinesterase (AChE) immunopositivity in the hippocampal CA1/CA2 and CA3 subregions and cerebral cortex.

Groups	CA1/CA2	CA3	Cortex
Control (G1)	1.16 ± 0.40 ^aA^	1.16 ± 0.40 ^aA^	0.83 ± 0.40 ^aA^
STZ (3 mg/kg, i.c.v.) (G2)	2.66 ± 0.51 ^bA^	2.83 ± 0.40 ^bA^	2.66 ± 0.51 ^bA^
Sham (citrate buffer, i.c.v.) (G3)	1.00 ± 0.00 ^aA^	1.16 ± 0.40 ^aA^	0.66 ± 0.51 ^aA^
GA (100 mg/kg/day) (G4)	2.00 ± 0.00 ^cA^	2.16 ± 0.40 ^cA^	1.83 ± 0.40 ^cA^
GA (200 mg/kg/day) (G5)	1.16 ± 0.40 ^dA^	1.33 ± 0.51 ^dA^	0.83 ± 0.40 ^dA^
EA (25 mg/kg/day) (G6)	1.83 ± 0.40 ^cA^	2.00 ± 0.00 ^cA^	1.83 ± 0.40 ^cA^
EA (50 mg/kg/day) (G7)	1.00 ± 0.00 ^dA^	1.16 ± 0.40 ^dA^	1.00 ± 0.00 ^dA^
Memantine (10 mg/kg/day) (G8)	2.00 ± 0.00 ^cA^	2.16 ± 0.40 ^cA^	2.00 ± 0.00 ^cA^

Data are presented as mean ± SD. Different lowercase superscript letters (a–d) indicate statistically significant differences between groups within the same column (*p* < 0.05). Different uppercase superscript letters indicate statistically significant differences between regions within the same group (*p* < 0.05), whereas the same uppercase letter indicates no significant regional difference.

**Table 4:** Overall mean semiquantitative in situ hybridization scores for tau, β-amyloid/APP-related, and acetylcholinesterase (AChE) mRNA signals in the hippocampal CA1/CA2 and CA3 subregions and cerebral cortex.

Groups	CA1/CA2	CA3	Cortex
Control (G1)	0.66 ± 0.51 ^aA^	0.66 ± 0.51 ^aA^	0.66 ± 0.51 ^aA^
STZ (3 mg/kg, i.c.v.) (G2)	2.66 ± 0.51 ^bA^	2.66 ± 0.51 ^bA^	2.33 ± 0.51 ^bA^
Sham (citrate buffer, i.c.v.) (G3)	0.66 ± 0.51 ^aA^	0.83 ± 0.40 ^aA^	0.66 ± 0.51 ^aA^
GA (100 mg/kg/day) (G4)	1.83 ± 0.40 ^cA^	1.83 ± 0.40 ^cA^	1.66 ± 0.51 ^cA^
GA (200 mg/kg/day) (G5)	1.00 ± 0.00 ^dA^	1.00 ± 0.00 ^dA^	0.83 ± 0.40 ^dA^
EA (25 mg/kg/day) (G6)	1.83 ± 0.40 ^cA^	1.66 ± 0.51 ^cA^	1.83 ± 0.40 ^cA^
EA (50 mg/kg/day) (G7)	1.16 ± 0.40 ^dA^	0.83 ± 0.40 ^dA^	1.00 ± 0.00 ^dA^
Memantine (10 mg/kg/day) (G8)	1.66 ± 0.51 ^cA^	1.83 ± 0.40 ^cA^	1.66 ± 0.51 ^cA^

Data are presented as mean ± SD. Different lowercase letters indicate statistically significant differences between groups within the same brain region (*p* < 0.05). Different uppercase letters indicate statistically significant differences among brain regions within the same group (*p* < 0.05), whereas the same uppercase letter indicates no significant regional difference.

## Data Availability

The original contributions presented in this study are included in the article/Supplementary Materials. Further inquiries can be directed to the corresponding author.

## Supplementary Materials

The following supporting information can be downloaded at: https://www.mdpi.com/article/10.3390/ph19091392/s1, Figure S1: Representative heatmaps of swimming patterns during the Morris water maze probe trial on Day 5. The platform was removed during the probe trial. The northwestern quadrant, which had previously contained the hidden platform, was designated as the target quadrant, and the circular marker indicates the former platform location. Each heatmap represents one animal from the corresponding experimental group and is provided as a qualitative visualization of swimming behavior. The experimental groups were as follows: G1, control; G2, STZ (3 mg/kg, i.c.v.); G3, sham (i.c.v. citrate buffer); G4, STZ + GA (100 mg/kg/day); G5, STZ + GA (200 mg/kg/day); G6, STZ + EA (25 mg/kg/day); G7, STZ + EA (50 mg/kg/day); and G8, STZ + MEM (10 mg/kg/day). GA, gentisic acid; EA, erucic acid; MEM, memantine hydrochloride; STZ, streptozotocin; i.c.v., intracerebroventricular; N, north; S, south; E, east; W, west. Figure S2: Representative MRM chromatograms and product-ion spectra for glutamic acid analysis in (A) blank, (B) G1 control, and (C) G2 STZ-treated hippocampal samples. Glutamic acid was monitored using the transitions *m*/*z* 147.8 → 129.9 and *m*/*z* 147.8 → 84.0. Chromatographic signals in the study samples corresponding to glutamic acid were observed at retention times of approximately 2.0–2.1 min. An ion-ratio tolerance of ±20% relative to the reference-standard response was used as the identification criterion. The shaded regions indicate the chromatographic areas selected for integration. The data should be interpreted together with the retention-time and ion-ratio acceptance criteria rather than as independent confirmation of analyte identity. The signal observed in the blank at 2.032 min did not meet the predefined ion-ratio acceptance criterion (observed deviation: 600.5%) and was therefore not identified as glutamic acid. G1, control group; G2, STZ-treated group; MRM, multiple reaction monitoring; STZ, streptozotocin.

## Abbreviations

Aβ	Amyloid-beta
AD	Alzheimer’s disease
AChE	Acetylcholinesterase
AEC	3-Amino-9-ethylcarbazole
ANOVA	One-way analysis of variance
APP	Amyloid precursor protein
ARRIVE	Animal Research: Reporting of In Vivo Experiments
CA1	Cornu ammonis 1
CA2	Cornu ammonis 2
CA3	Cornu ammonis 3
DIG	Digoxigenin
EA	Erucic acid (cis-13-docosenoic acid)
EAAT	Excitatory amino acid transporter
ESI	Electrospray ionization
GA	Gentisic acid (2,5-dihydroxybenzoic acid)
H&E	Hematoxylin and eosin
HRP	Horseradish peroxidase
i.c.v.	Intracerebroventricular
icv-STZ	Intracerebroventricular streptozotocin
IHC	Immunohistochemistry
IQR	Interquartile range
ISH	In situ hybridization
LC–MS/MS	Liquid chromatography–tandem mass spectrometry
LOD	Limit of detection
LOQ	Limit of quantification
MEM	Memantine hydrochloride
MRM	Multiple reaction monitoring
MWM	Morris water maze
NMDA	N-methyl-D-aspartate
PBS	Phosphate-buffered saline
PPAR-δ	Peroxisome proliferator-activated receptor delta
ROS	Reactive oxygen species
RSD	Relative standard deviation
STZ	Streptozotocin
tau	Microtubule-associated protein tau
VGLUT	Vesicular glutamate transporter
