# Supplementary material for: Early Neuroprotective Effects of Erucic Acid and Gentisic Acid on Hippocampal Glutamate Concentrations and Alzheimer-like Molecular Alterations in an Intracerebroventricular Streptozotocin Rat Model
Source: Pharmaceuticals (Basel). 2026 Sep 2;19(9):1392. doi: 10.3390/ph19091392 (PMC13610910; doi:10.3390/ph19091392)
Supplement: Supplementary file 1 [file pharmaceuticals-19-01392-s001.zip › pharmaceuticals-4512199-supplementary.pdf]

Supplementary Materials

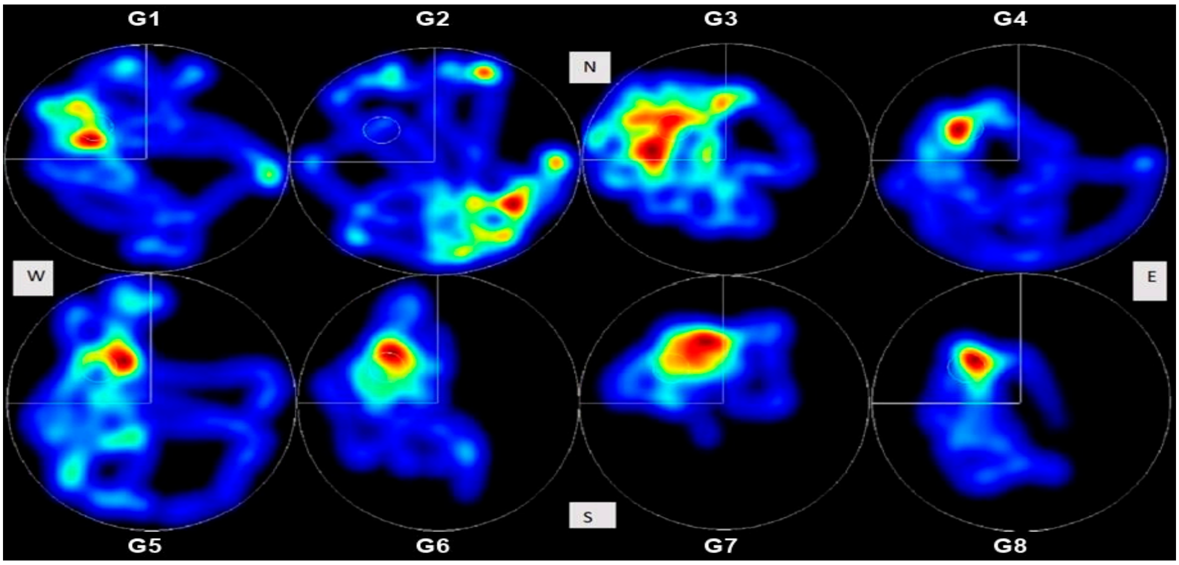

Supplementary Figure S1. Representative heatmaps of swimming patterns during the Morris water maze probe trial on Day 5. The platform was removed during the probe trial. The northwestern quadrant, which had previously contained the hidden platform, was designated as the target quadrant, and the circular marker indicates the former platform location. Each heatmap represents one animal from the corresponding experimental group and is provided as a qualitative visualization of swimming behavior. The experimental groups were as follows: G1, control; G2, STZ (3 mg/kg, i.c.v.); G3, sham (i.c.v. citrate buffer); G4, STZ + GA (100 mg/kg/day); G5, STZ + GA (200 mg/kg/day); G6, STZ + EA (25 mg/kg/day); G7, STZ + EA (50 mg/kg/day); and G8, STZ + MEM (10 mg/kg/day). GA, gentisic acid; EA, erucic acid; MEM, memantine hydrochloride; STZ, streptozotocin; i.c.v., intracerebroventricular; N, north; S, south; E, east; W, west.

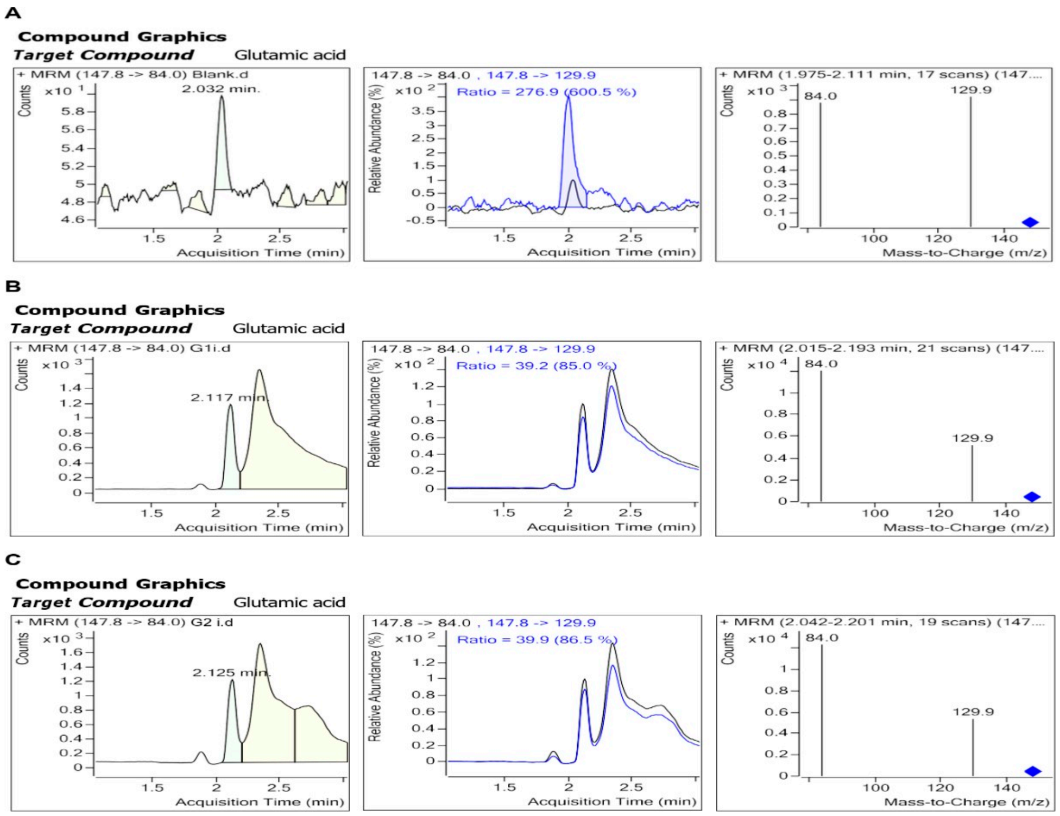

Supplementary Figure S2. Representative MRM chromatograms and product-ion spectra for glutamic acid analysis in (A) blank, (B) G1 control, and (C) G2 STZ-treated hippocampal samples. Glutamic acid was monitored using the transitions m/z 147.8 → 129.9 and m/z 147.8 → 84.0. Chromatographic signals in the study samples corresponding to glutamic acid were observed at retention times of approximately 2.0–2.1 min. An ion-ratio tolerance of ±20% relative to the reference-standard response was used as the identification criterion. The shaded regions indicate the chromatographic areas selected for integration. The data should be interpreted together with the retention-time and ion-ratio acceptance criteria rather than as independent confirmation of analyte identity. The signal observed in the blank at 2.032 min did not meet the predefined ion-ratio acceptance criterion (observed deviation: 600.5%) and was therefore not identified as glutamic acid. G1, control group; G2, STZ-treated group; MRM, multiple reaction monitoring; STZ, streptozotocin.
